# The ChIP-Seq tools and web server: a resource for analyzing ChIP-seq and other types of genomic data

**DOI:** 10.1186/s12864-016-3288-8

**Published:** 2016-11-18

**Authors:** Giovanna Ambrosini, René Dreos, Sunil Kumar, Philipp Bucher

**Affiliations:** 1 School of Life Sciences, Ecole Polytechnique Fédérale de Lausanne (EPFL), CH-1015 Lausanne, Switzerland; 2 Swiss Institute of Bioinformatics (SIB), CH-1015 Lausanne, Switzerland

**Keywords:** ChIP-seq data analysis, Bioinformatics resources, Web server, Peak finding, Genomic context analysis, Transcription factor binding sites, Histone modifications, DNA sequence motifs

## Abstract

**Background:**

ChIP-seq and related high-throughput chromatin profilig assays generate ever increasing volumes of highly valuable biological data. To make sense out of it, biologists need versatile, efficient and user-friendly tools for access, visualization and itegrative analysis of such data.

**Results:**

Here we present the ChIP-Seq command line tools and web server, implementing basic algorithms for ChIP-seq data analysis starting with a read alignment file. The tools are optimized for memory-efficiency and speed thus allowing for processing of large data volumes on inexpensive hardware. The web interface provides access to a large database of public data. The ChIP-Seq tools have a modular and interoperable design in that the output from one application can serve as input to another one. Complex and innovative tasks can thus be achieved by running several tools in a cascade.

**Conclusions:**

The various ChIP-Seq command line tools and web services either complement or compare favorably to related bioinformatics resources in terms of computational efficiency, ease of access to public data and interoperability with other web-based tools. The ChIP-Seq server is accessible at http://ccg.vital-it.ch/chipseq/.

**Electronic supplementary material:**

The online version of this article (doi:10.1186/s12864-016-3288-8) contains supplementary material, which is available to authorized users.

## Background

**Fig. 1:**
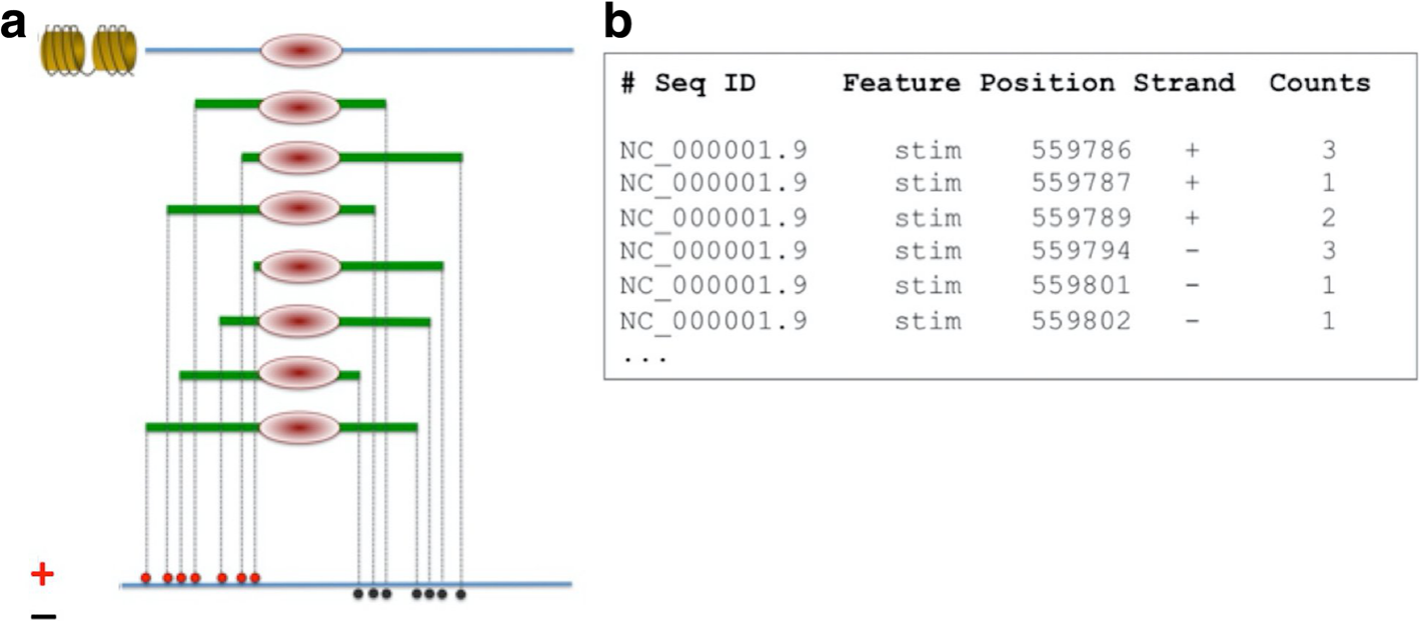
ChIP-seq assay and data representation. **a** Schematic representation of a ChIP-seq experiment. Chromatin is first crosslinked and cut into small pieces. DNA fragments bound by a specific protein are isolated with an antibody and sequenced from the ends using a short-read sequencing technology. The reads are then computationally mapped to the genome. Note that the reads mapping to the plus + and – strand of the genome, respectively, are expected to form clusters upstream and downstream of the protein binding site. **b** ChIP-seq data representation in SGA format. SGA is the working format of the ChIP-Seq tools. Each line contains five obligatory fields: sequence identifier (here an NCBI RefSeq ID), feature name (designating a ChIP-seq experiment), sequence position, strand and read count. Note that only the genomic position corresponding to the 5′end of the mapped sequence read is recorded in an SGA file

The advent of chromatin immunoprecipitation combined with sequencing (ChIP-seq) has revolutionized research in gene regulation. A basic ChIP-seq experiment is schematized in Fig. 1a. In essence, the technology allows mapping of in vivo DNA-protein interactions at very high resolution on a genome-wide scale and at low cost [1, 2]. Thanks to ChIP-seq, the tissue-specific chromatin state of gene regulatory regions has become visible and transcription factor (TF) binding events leading to expression changes of target genes can directly be observed. Unsurprisingly therefore, this technique has become a standard assay for genomics research in very short time. A wealth of data has been released over the last years, which is of potential interest to any biologist working on gene regulation. Noteworthy in this context are the reference data sets released by the large international consortia ENCODE [3] and Roadmap Epigenomics [4] providing detailed epigenetic characterization for a large number of tissues and cell types from human and mouse.

Being low cost and high-throughput, the true bottleneck of a ChIP-seq experiment is data analysis. Although the bioinformatics community has reacted quickly to this new challenge by developing a wealth of specialized software tools, we believe that ChIP-seq data are still largely under-analyzed. Lots of new insights into gene regulatory mechanisms could be gained by simply analyzing existing data under new angles. Likewise, such data should be used to corroborate or refute biological hypotheses put forward in papers. Consequently there is a need for bioinformatics resources providing easy access to public ChIP-seq data to a wider research community and offering efficient software tools for integrative analysis of large data sets.

Reviews on ChIP-seq data analysis can be found in [5, 6]. A typical analysis pipeline starts with the mapping of sequence reads to the genome of the corresponding species followed by peak finding, peak annotation and DNA motif analysis. The publicly available resources for carrying out these tasks have been implemented in different ways: (i) as stand-alone UNIX command-like tools, e.g. Bowtie [7] for read alignment and MACS [8] for peak finding, (ii) as R packages, e.g. Rolexa [9] and metagene [10], (iii) as wrapper shell scripts executing a comprehensive analysis pipeline by calling multiple external programs in a cascade [11], (iv) as integrated software platforms with a graphical interface requiring local installation, e.g. Homer [12], cisGenome [13] and seqMiner [14], and (v) as web-servers, e.g. Cistrome [15].

Here we present the ChIP-Seq tools, a collection of programs implementing a variety of ChIP-seq data analysis algorithms downstream of read mapping. We initially developed these programs for our own research, *see* [16] for an early application. Encouraged by positive feedback from our collaborators, we later invested considerable efforts to make our tools available to a wider community.

The ChIP-Seq tools are made available in two forms serving different user communities: as an open source collection of Unix command-line programs and as an interactive web interface. The latter is tightly integrated with other web-based resources maintained by our group, including the Eukaryotic Promoter Database EPD [17], and the Signal Search Analysis (SSA) server [18]. We believe that the ChIP-Seq tools fill an important niche in computational genomics due to their original design and unique capabilities. More than any competing program package we know of, our tools are streamlined for memory efficiency and speed, enabling researchers to process high volumes of data on modest computer hardware. With the ongoing data explosion resulting from the fact that sequencing costs are going down faster than data processing costs, this aspect may even become appealing to large sequencing centers. The ChIP-Seq web server offers access to a large database of uniformly formatted ChIP-seq and other types of genomics data, covering a broad range of organisms from yeast to human, making it an interesting web resource for bioinformaticians involved in large-scale comparative studies of epigenetic profiling data from different species and tissues. Biologists primarily interested in analyzing their own samples have the possibility to compare their data with analogous data from other studies and to complement their analyses with data covering other aspects of chromatin structure and function.

The remainder of the paper is organized as follows. The Implementation Section explains the design principles of the ChIP-Seq command line tools and briefly introduces the most important programs. It further includes a description of the database of public genomics data sets installed at the backend of the web server. The Results Section presents the web interface to the ChIP-Seq tools, including a brief description of connected web resources developed by our group. The capabilities of the ChIP-Seq server are illustrated on typical examples in a tutorial style fashion. (Step-by-step instructions for reproducing the results and Figures are given in Additional file 1.) The Results Section concludes with a comparison of the ChIP-Seq tools to similar resources. The Conclusions Section briefly recapitulates the hallmarks and highlights of our resource and ends with an outlook on future developments.

## Implementation

### Design principles of the ChIP-Seq command-line tools

The ChIP-Seq command-line tools package is a collection of stand-alone programs written in C and Perl. The package is of highly modular design. Each program reads from standard input, writes to standard output, and carries out a well-defined elementary operation. Standard ChIP-seq data analysis tasks such as peak finding are often accomplished by sequentially running multiple program units in a UNIX pipe.

The logical design of the ChIP-Seq tools is based on a standard input-output format called SGA (Fig. 1b). SGA stands for “Simple Genome Annotation”. All ChIP-Seq programs take input in SGA format and many produce output in SGA format as well. SGA is a tab-delimited text format with five obligatory fields per line: chromosome (sequence) identifier, feature, position, strand, and counts. Additional fields may follow but are not essential and thus ignored by most ChIP-Seq tools programs.

SGA, unlike BED or GFF, is a single position format that assigns positive integer values (counts field) to selected bases of the genome. The format can represent any type of experimental data or genome annotations compatible with these restrictions. In the case of ChIP-seq data, the position and strand fields refer to the base that corresponds to the 5′-end of a mapped sequence read, and the count field indicates how many reads were mapped to this same position. Other data types that can be represented by SGA and processed with ChIP-Seq tools programs include mapped sequence reads from DNase-seq or MNase-seq assays, CAGE tags, transcription start sites from genome annotation databases (e.g. ENSEMBL) or SNPs from the 1000 Genomes Project [19].

There are other specificities of the SGA format which are worthwhile mentioning. The strand field may be set to zero (rather than “+” or “–”) to indicate that the genomic feature is intrinsically unoriented. The feature field serves to distinguish lines from different data sources or representing different genomic features, which is necessary because many ChIP-Seq tools programs require two features as input while physically accepting only one input file. Perhaps the truly mission-critical requirement of the SGA format is that lines must be sorted by chromosome, position and strand. Once sorted, the data can be processed using fast algorithms that produce results by one pass through the genome. As a consequence, all ChIP-Seq tools programs have time complexity *O*(*N*), *N* being the number of lines in the input file. The sorting also enables programs to read in and process data for only one chromosome at a time, resulting in a gain in memory efficiency.

The ChIP-Seq package currently comprises 21 stand-alone programs (in Additional file 1: Table S1), many of them performing mere reformatting or preprocessing tasks. In the following, only the programs addressing area-specific non-trivial tasks such as peak finding will be described in more detail. Algorithms requiring substantial computations are implemented in C, others are currently offered as Perl scripts. In the long run, we plan to replace all Perl scripts by C programs. The major algorithms are given as pseudo-code in Additional file 1: Text S3.

### Feature correlation tools

The hallmark of this class of programs is that they take two types of genomic features as input and generate numerical data suitable for graphical display or follow-up analysis by stats packages such as R. The two features are supplied in a single SGA file, typically generated on the fly via a sort-merge operation and then passed to the program via standard input.

*Chipcor* is the prototype feature correlation tool. To describe how it works, let’s call the two features “reference” and “target”. *Chipcor* then computes a profile (numerical vector) indicating the average abundance of the target feature at various distances upstream and downstream of the reference feature. The user has to specify the distance range around the reference feature and size of the bins in which target features are to be counted. The output of *chipcor* is a tab-delimited text file which can be visualized as so-called aggregation plot (AP) [20].

*Chipcor* is used for two main purposes: generation of cross-correlation plots and genomic context analysis. In the former case, tags mapped to the + and – strands from the same ChIP-seq experiment are supplied as reference and target features respectively. The resulting profile reveals the average length of the pulled-down fragments and is essentially equivalent to the cross correlation plot recommended by the ENCODE consortium [21] for quality control purposes.

Genomic context analysis serves to visualize positional correlations between features of different type. The reference feature typically consists of a relatively small set of genomic positions originating from manual annotation efforts (e.g. a promoter collection) or automatic processing of primary experimental data (e.g. a ChIP-seq peak list). The target feature may be of any kind including high density features such as mapped sequence reads from ChIP-seq experiments. Many examples of feature correlation plots can be found in the Results Section further below.

There are two other programs taking two feature types as input and performing similar operations as *chipcor*. The first one, *chipextract,* extracts target feature counts in binned genomic regions for each reference feature individually. The output is an integer matrix which can be visualized as a heat map. The second program is *chipscore* that counts target features within a user-defined region around the reference features (without splitting the region into smaller bins). The output is an extended SGA file composed of the reference feature lines of the input file and an additional field presenting the target features counts in the specified regions. The program can also be used as a feature selection tool by specifying a non-zero tag threshold via a command line option. Unlike most other ChIP-Seq tools programs, *chipscore* reads all optional fields from reference feature lines and transfers them to the output, which makes it possible to annotate the same reference feature set with tag counts from multiple experiments.

### Peak finders and segmentation tools

The ChIP-Seq tools include two programs to search ChIP-seq data for signal-enriched regions. *Chippeak* is a classical peak finder appropriate for finding transcription factor binding sites. *Chippart* is a segmentation tool or “broad peak” finder that partitions the genome into signal-enriched and depleted regions. *Chippart* is typically used for processing histone modification profiles. In terms of input-output behavior, the main difference between the two programs is that *chippeak* returns a single genomic position corresponding to the peak center whereas *chippart* returns the start and end positions of genomic regions.

*Chippeak* is a basic peak finder. The current version takes a single feature type as input and thus cannot make use of a control experiment for local background correction. Conceptually, *chippeak* uses a sliding window approach to identify signal enriched regions. The numbers of tags are counted in a window of fixed size along the genome and peaks correspond to local maxima in terms of tag coverage. The peak-calling process is primarily controlled by three parameters: the *window width*, the *tag threshold*, i.e. the minimal number of tags a window must contain in order to qualify as a peak, and the *vicinity range*, i.e. the size of the region within which the window must be a local maximum. Speed is gained by restricting the search to positions hit by at least one ChIP-seq tag. The original position corresponding to a mapped sequence tag can optionally be replaced by a “weighted average position” computed from the tag distribution within the corresponding window. *Chippeak* is at least 10 times faster than any other ChIP-seq peak finder we have tested (Additional file 1: Table S3). In spite of its simplicity, it generally performs well, sometimes even better than competing programs using a more elaborate statistical model to assess the significance of a peak [22, 23].

*Chippart* partitions the genome into an alternating series of signal-enriched and signal-depleted regions. Signal strength is defined as counts per base-pairs. The output is a so-called “regions” SGA file, a special type of SGA file where each line marks a segment boundary. Lines with a “+” sign in the strand filed mark the beginning, those with a “−” sign mark the end of an enriched segment. The total number of counts in an enriched region is returned in the fifth field. The optimal segmentation of a chromosome is found by means of an efficient dynamic programing algorithm maximizing a global segmentation score (for details, *see* pseudo-code in Additional file 1: Text S3). This score critically depends on two parameters, the count density threshold which defines the border between enriched and depleted, and the transition penalty which controls the extent of fragmentation of the genome (high penalty favors large regions).

### Preprocessing programs and command line options

*Chipcenter* is used for centering mapped sequence tags from a ChIP-seq experiment, a preprocessing step that is also referred to as tag shifting [24]. In essence, the tags assigned to the “+” strand of the genome are shifted by a user-defined distance downstream, those assigned to the “−” strand are shifted by the same distance upstream. The output is a centered SGA file with the strand field set to zero on all lines. Centering is motivated by the expected distribution of sequence tags around ChIP-seq targets (Fig. 1a) and increases the positional resolution of the ChIP-seq signal.

The program *counts_filter* eliminates lines from an input SGA file which fall within “blacklisted” genomic regions. Input is a composite SGA file containing two types of genomic features, one that is subject to filtering and will be transferred to output, the other one defining the start and end points of the blacklisted regions. This program is often used to eliminate ChIP-seq tags of peaks that fall within annotated repeat regions.

Each ChIP-Seq program is controlled by a number of command line options. Two of these options are common to many programs and thus deserve brief mentioning. Option *–c* (count cut-off) specifies the maximal number of counts accepted at a single genomic position. If set to one, it has the same effect as eliminating duplicates at the read mapping step. Option –o (oriented) controls the interpretation of the strand field. For programs like *chipcor*, which take two feature types as input, it has the effect that the target features are processed in “reverse-complementary” manner for reference features assigned to the “–” strand. Reverse-complementary means that a target feature assigned to the “+” strand and located 100 bp upstream of the reference feature would be treated like a target feature assigned to the “−” strand and located 100 bp downstream. For *chippeak*, the oriented mode has the effect that peak finding is carried out separately for the “+” and “–” strand of the genome. This behavior is useful, for instance, for finding clusters of TSSs in CAGE data.

### The mass genome annotation (MGA) repository

The abbreviation MGA has been introduced by the DDBJ and EMBL nucleotide sequence data libraries and stands for “Mass sequences of Genome Annotation” [25]. We have adopted this term to designate the data repository harboring all public data that can be accessed and analyzed through our web servers. The MGA repository is also accessible via anonymous FTP. Though not maintained by a database management software, the MGA repository is highly structured and respects rigorous formatting standards. All data sets are presented in SGA format. Chromosomes and contigs are invariantly identified by versioned RefSeq [26] accession numbers (e.g. NC_000001.10 for chromosome 1 of human genome assembly GRCh37/hg19). This eliminates the risk of producing wrong results by comparing sequence positions relating to different versions of the same chromosome.

The MGA repository is hierarchically split into subdirectories. There is one root-level subdirectory for each supported genome assembly. Data sets from the same study are organized as series, as in GEO [27]. All data files pertaining to the same series are kept in the same leaf-level subdirectory together with a manually edited documentation file in HTML format. Each series contains two additional machine-readable text files, one providing information about the series as a whole, the other one about individual samples. These files are primarily used by software components of the web server. For instance, the data access menus of the program input forms are automatically generated in this way. The series documentation files are web-accessible via a hierarchically organized table with expandable subsections.

The MGA repository currently contains more than 10’000 data samples, about half of them from ChIP-seq and other chromatin profiling assays such as MNase-seq [28] and DNase-seq [29]. High priority is also given to TSS mapping data such as CAGE [30] which constitute the source data for EPDnew [17], the automatically compiled part of the Eukaryotic Promoter Database EPD. More than 1500 tissue and cell-type specific TSS libraries from ten different species are available. An overview of the current contents of the MGA repository is shown in Additional file 1: Table S2.

Unlike other bioinformatics resources which harbor large public next generation sequencing (NGS) data collections such as Cistrome, the MGA repository also provides a large variety of non-experimental, computationally derived or manually generated data sets. Examples are transcription start and end site (TSS, TES) collections from ENSEMBL [31], lists of repeated elements from Repbase [32], single nucleotide polymorphisms from dbSNP [33] and cross-genome conservation scores from the UCSC Genome Browser database [34]. Computationally derived features include published ChIP-seq peak lists, which we offer in addition to the read mapping data if available. Some genomic feature lists are provided in extended SGA format with an optional sixth field containing a gene name (TSS lists), or a statistical significance score (peak lists).

The majority of the NGS data were downloaded from GEO, ArrayExpress [35] or the UCSC Genome Browser database. A few (mostly older) data sets were directly downloaded from the author’s institutional websites. If available, we used libraries of already mapped sequence tags in BAM or BED format as source data. A technical description of the conversion procedure from BAM or BED to SGA is given in Additional file 1: Text S2. Otherwise, we carried out the tag-to-genome mapping ourselves, usually starting from FASTQ files and using Bowtie [36] for read alignment. The conversion procedures and the URLs for the source data are given in the corresponding documentation files for each series.

Some data sets in the MGA repository have undergone substantial modifications relative to the original file, either to reduce the size or to make the representation compatible with an integer-based single-position format. As mentioned before, the repeat libraries used for repeat masking are provided as regions SGA files. For genomic conservation analysis, the MGA offers compacted versions of the phastCons [37] and phyloP [38] tracks from the UCSC Genome Browser database to speed up the analysis at the expense of some precision. As a general rule, we try to keep the size of SGA files below 100 million lines.

The machine-readable series description file contains the complete path to the data directory, a descriptive title, a literature or database reference in textual form plus GEO, ArrayExpress and/or PubMed IDs, if available. The ChIP-Seq web server transfers these fields to output pages in order to give appropriate credit to the authors of the data and to generate hyperlinks to external resources.

The sample description file provided for each series is a tab-delimited table with lines corresponding to samples. It contains essential information for the ChIP-Seq server as well as command-line users. The first field of each line contains the name of the corresponding SGA file and is followed by fields containing a sample description and the feature name used in the SGA file. The fourth field assigs each sample to a so-called “data type.” Examples of data types are “ChIP-seq”, “ChIP-seq-peak” or “Genome Annotations”. Note that a series may contain samples belonging to different data types. Additional fields indicate whether the feature is “oriented” and whether the sample is provided in FPS format (FPS stands for “functional position set” and refers to the native format of the SSA package, *see* below). GEO or ArrayExpress IDs are also included if applicable.

An SGA file is called “oriented” if the strand field is occupied by + and – signs; “unoriented” SGA files have the strand field invariantly filled with zeros. Small data sets comprising less than 100’000 genomic positions (typically genome annotations and peak lists) are usually provided in both SGA and FPS format. As a consequence, these files are also accessible through the input server menus of the SSA server.

The MGA repository currently contains NGS data for ten species, all mapped to a single, so-called primary assembly of the corresponding species, except for human where the data are split over the assemblies hg18 and hg19 (in Additional file 1: Table S2). In addition, basic support files (gene annotations, repeat libraries, and conservation tracks) are provided for additional assemblies of the same species. These files offer users who have data mapped to a non-primary assembly an easy way to carry out a certain number of standard analysis tasks, such as repeat masking, analyzing histone marks in promoter regions, or generating sequence conservation profiles for ChIP-seq peaks. Using the data from the main assembly would require remapping of the genomic coordinates in the original file (which is pretty straightforward as well). Command line users can remap SGA files with the aid of the SGA-to-BED conversion utilities from the ChIP-Seq command-line tools and the liftOver utility from the UCSC Genome Browser [39]. The ChIP-Seq web interface allows remapping of genomic coordinates upon data upload.

## Results

### General characteristics of the ChIP-Seq web server

The ChIP-Seq server is a web interface to the ChIP-Seq tools and the MGA repository. Any result that can be produced over the web interface can also be produced from the command line with data files downloaded from the MGA repository. For the sake of transparency and reproducibility, the UNIX shell scripts executed by the server are posted on all results pages. These scripts may also serve as templates to command-line users who would like to integrate the web-based applications into a data analysis workflow running on a local computer.

Note that throughout this manuscript, web interfaces to command tools will be spelled with a dash in the middle followed by the capitalized tool name, e.g. ChIP-Cor is the web interface of *chipcor.*

Like the stand-alone programs, the individual web services are designed to be modules that can be used sequentially. The output from one application can be transferred to the next application via direct navigation buttons. However, there is no one-to-one correspondence between command-line programs and web applications. A web application typically fulfils a more complex task requiring the execution of several programs and scripts by the server. For instance, the ChIP-Peak web application allows users to do tag centering with *chipcenter,* repeat-masking with *counts_filter,* peak finding with *chippeak* and conversion of the output SGA file into BED format in a single run. Analogous web applications exist for the major ChIP-seq programs *chipcor*, *chipextract* and *chippart. Chipscore* is offered via a follow-up menu displayed on the Chip-Cor results page. The application ChIP-Convert serves as a hub for data input, format conversion, and data export to other applications.

### Input forms

**Fig. 2:**
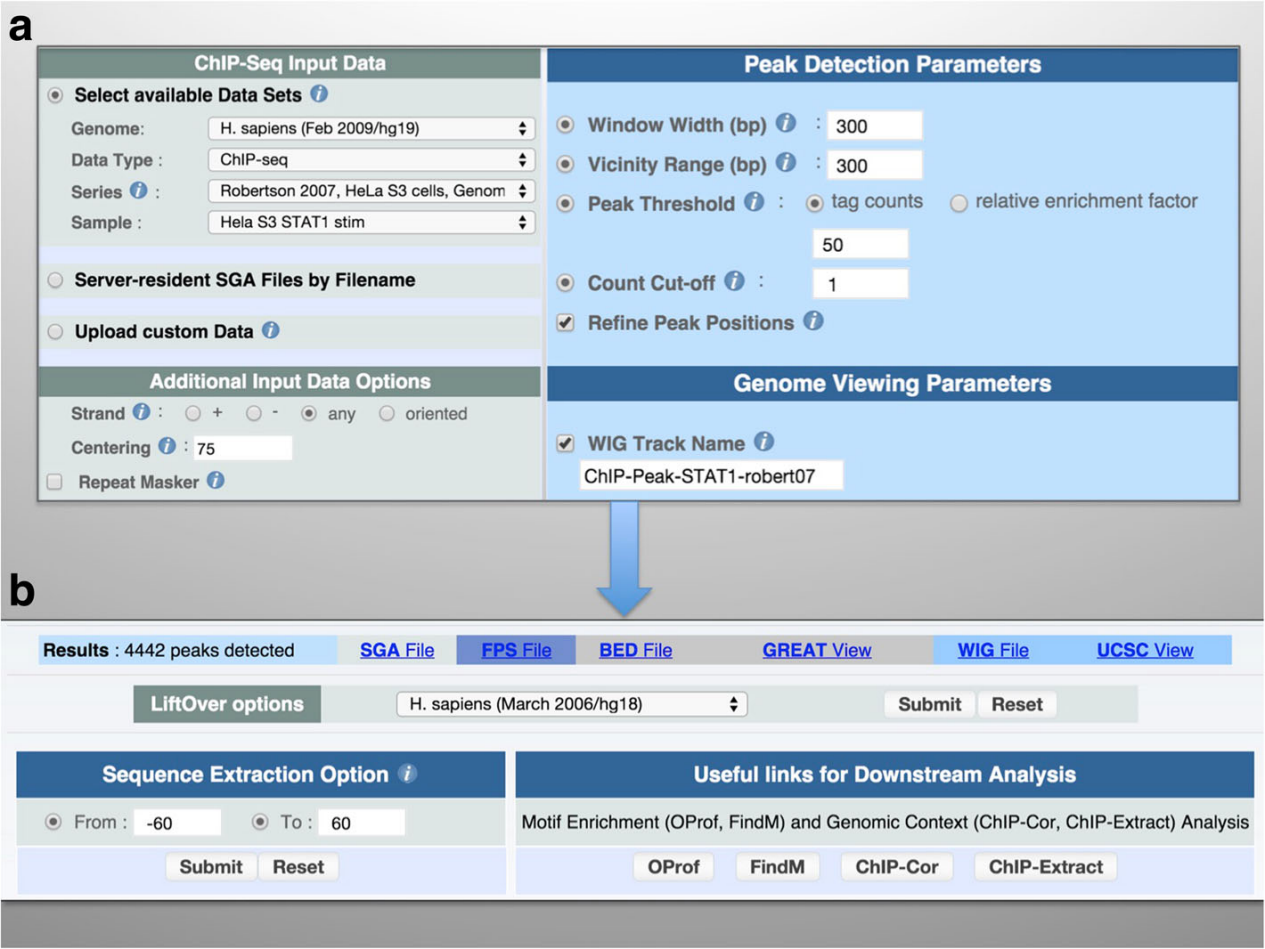
Web interface of ChIP-Peak. **a** Input form. The inputs correspond to the example presented in this paper. A server-resident ChIP-seq sample from the MGA repository has been selected through the data access menu. Alternately, users could upload their own data by clicking on the “Upload custom data” radio button. **b** Output page. The peak list can be downloaded in various formats. Hyperlinks are provided for sending the peak list directly to external servers for peak annotation. The “Sequence Extraction Option” enables users to extract sequences around the peak centers in Fasta format. Direct navigation buttons enable downstream analysis with other tools from the ChIP-Seq and SSA servers

The input forms have a standard design, with three parts: one for data access, one for specifying preprocessing options, and one for entering the analysis parameters of a particular application (Fig. 2a).

The input data can be specified or uploaded in three ways: (i) through a hierarchical menu that provides access to the MGA repository, (ii) by specifying the name of a server resident file and (iii) by uploading a data file from a local computer or via URL.

The menu-driven access to the MGA repository has four hierarchical levels. At the first level, the user can choose one of the supported genome assemblies. The second level is organized by data type (ChIP-seq, RNA-seq, genome annotations, etc. *see* previous Section). Large data collections from international consortium projects are provided as separate data types. The two lower levels correspond to series and samples. Note that the server menu is not a perfect mirror of the MGA directory structure. A MGA series may contain samples of different data types, in which case the series name appears multiple times under different data types in the server menus.

Data access via filenames allows developers and informed collaborators to access server-resident files via a local filename and directory path. This mechanism is further used for transferring data between web applications. If a user clicks on a direct navigation button, the name of the temporary file generated by the previous application will appear in the input form of the next application.

External files can be uploaded in various genome annotation formats, either from a local data directory or via a URL. Large files can be transferred in compressed form (zip and gzip). In principle, the server accepts any kind of sequence identifier relating to any species. However, if the identifiers do not correspond to a supported genome assembly, many useful features will not be available. For instance, it will not be possible to analyze the uploaded data jointly with data from the MGA repository. Direct navigation buttons to the motif analysis programs from the SSA server will also be suppressed. If data correspond to a supported genome assembly, UCSC-style chromosome names or contig identifiers have to be used and the corresponding assembly needs to be specified on the input form. Alternatively, chromosomes can be identified by versioned RefSeq accession numbers. RefSeq identifiers are always used internally by the server.

For reasons of efficiency, we recommend uploading data in SGA format. However, all major applications support the following other input formats: BED, BAM, GFF and FPS. There is a dedicated application named ChIP-Convert for importing and converting external data formats into compressed SGA. Once converted, an uploaded data set will be accessible via URL or a temporary name for at least one hour. For repeated use over a certain period of time, we recommend reformatting voluminous data files into compressed SGA via ChIP-Convert, rather than repeatedly using conversion options provided by program-specific input forms. ChIP-Convert also provides more specific conversion schemes such as proper conversion of the BED-like narrowPeak format used by ENCODE.

All input forms are pre-loaded with reasonable default parameters. Importantly, in the case of the peak-finding applications ChIP-Peak and ChIP-Part, the critical threshold parameter is expressed as a fold-change over the average count density of the input sample, resulting in robust and reproducible behavior across data sets.

### Results pages and interoperability with other tools

The output pages generated by the ChIP-Seq server are also standardized (Fig. 2b). For programs that produce SGA output, the results are provided in BED and FPS format as well if the input relates to a supported genome assembly. If this is the case, a number of additional action buttons will be displayed on the output pages, including a link for viewing the results in a UCSC Genome Browser window, a menu that enables the user to extract sequences within a specified range around the genomic positions, and several buttons that will directly upload the results (genomics coordinates) to other ChIP-Seq server applications or external web services. For web applications that produce numerical output, the results consist of a graphical representation plus a download button for saving the numbers in text file format. For scientific articles, it is often desirable to combine results from several web jobs in a single figure (as it is done here). This can be achieved by saving the numerical results files to disk and re-importing them into the user’s preferred graphics program.

The ChIP-Seq server is tightly interconnected with other resources developed by our group, including the Eukaryotic Promoter Database (EPD), the Signal Search Analysis (SSA) server and the recently introduced PWMTools. Of particular relevance to ChIP-Seq data analysis is the SSA server, a suite of programs for the discovery and characterization of DNA sequence motifs that occur in the vicinity of a functional genomic site [40]. These programs were developed during the eighties primarily for the purpose of analyzing eukaryotic promoters. With the advent of ChIP-seq, the SSA programs have become useful in a new context, namely for the analysis of DNA motifs that are enriched near peak center positions. The most relevant tools are OProf (Occurrence Profile) and FindM (Find Motif).

OProf takes as input a list of genome positions plus a DNA motif definition, and returns a “motif occurrence profile”. A DNA motif can be entered as a IUPAC consensus sequence, as a position weight matrix (PWM) or, alternatively, taken from a public PWM collection via a pull-down menu. Currently, more than 2000 TF binding specificity matrices are offered to the user. OProf is analogous to ChIP-Cor, the main difference being that the target features, i.e. the motif matches, are computed on the fly by the program rather than read from an input file.

FindM takes the same input as OProf but produces a different kind of output, namely a new list of genome positions. The program may be used to select genomic positions from an input file which are (or are not) flanked by a given motif within a certain distance range. Alternatively, it can be forced to return the positions of flanking motifs. In ChIP-seq data analysis, FindM is sometimes used to realign computationally identified peaks on the exact base positions of the cognate TF binding motifs.

The PWMTools server provides access to more recently developed PWM-oriented software that uses SGA as working format. Two applications, which are potentially useful for ChIP-seq data analysis, will be described briefly. PWMScan enables users to scan a whole genome with a PWM and returns a complete list of PWM matches in various formats. Such a list could then be uploaded to ChIP-Cor in order to extract those matches that are not occupied by the corresponding TF in vivo according to a ChIP-seq experiment. Genomic context analysis could be employed to find an explanation why these sites are not occupied in vivo*.*

PWMEval is a tool to assess the quality of a PWM based on its ability to distinguish in vivo TF binding sites from random genomic sequences. It takes as input a PWM plus a ranked ChIP-seq peak list and returns a ROC area under the curve (AUC) value as a performance measure. The affinity of a peak region to the PWM is computed as described in [41]. Note that the performance indicated by PWMEval depends equally on the good quality of the PWM and the ChIP-seq peak list. The tool can thus vice-versa be used for peak list quality assessment.

Interoperability between the ChIP-Seq server and external bioinformatics servers is assured through the BED format. Direct navigation buttons allow forwarding of BED output to the UCSC Genome Browser and optionally to the GREAT server [42] if the output file contains less than 20’000 lines. For peak annotation on a Galaxy-based platform such as Nebula [43], the output from ChIP-Peak can be transferred by right-clicking on the BED download button and using “Copy link location” to paste the URL into the file upload page of the Galaxy interface. The same mechanism can be used in the opposite direction. For instance, some researchers may prefer to do peak finding with MACS on a Galaxy server and then use ChIP-Cor to explore the genomic context of the peak regions on our servers. For motif finding with an external tool, e.g. MEME-ChIP [44], sequences can be extracted in FASTA format from all applications that produce genomic position lists as output.

### An example illustrating the use of the ChIP-Seq server

In the following, we are going to illustrate the capabilities of the ChIP-Seq server using data from an early landmark paper reporting the genome-wide mapping of STAT1 binding sites in interferon-γ stimulated HeLa cells [45]. The data set, which comprises about 15 million mapped sequence tags, is available from the ChIP-Seq server menu (Fig. 2a). We will also use a control data set from a ChIP-seq experiment done with unstimulated HeLa cells, were the STAT1 protein is supposed to reside in the cytoplasm and thus unable to bind to its target sites in the genome. The example we present is partly based on a tutorial presented elsewhere [46].

**Fig. 3:**
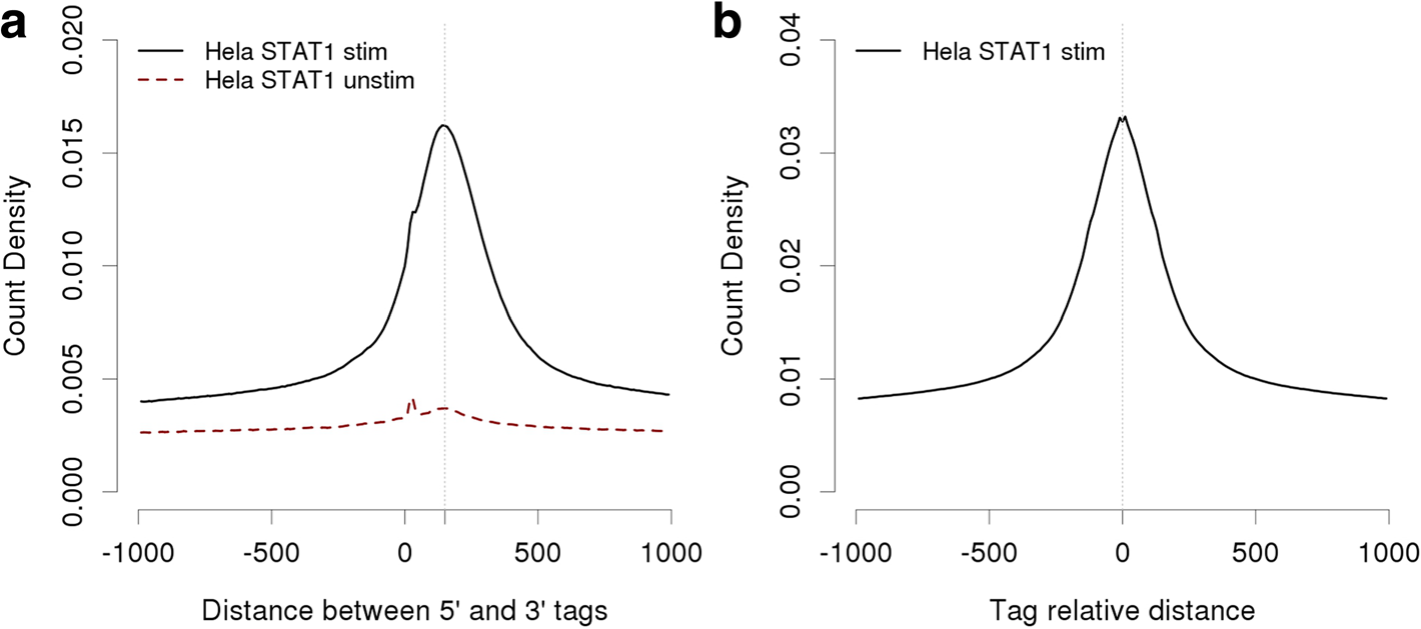
5′-3′end correlation and autocorrelation plots. **a** 5′-3′ end correlation plot for STAT1 ChIP-seq tags from interferon-γ stimulated and unstimulated HeLa cells. The *horizontal* position of the peak maximum suggests an average fragment size of about 150 bp. **b** Autocorrelation plot of 75 bp-centered STAT1 ChIP-seq tags from stimulated cells

**Fig. 4:**
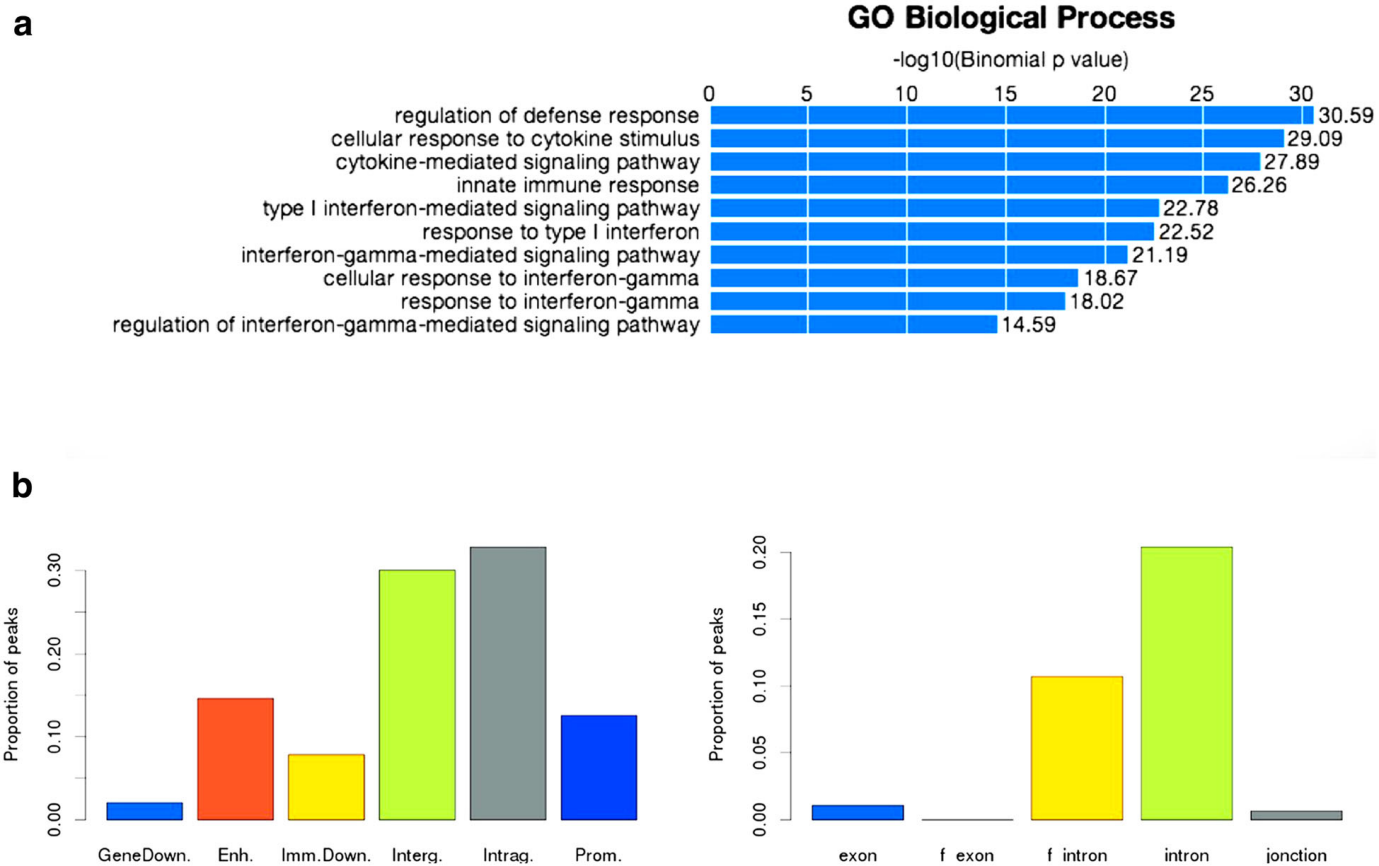
STAT1 peak annotation with external tools. **a** GO term enrichment analysis with GREAT. **b** Peak location statistics with Nebula

**Fig. 5:**
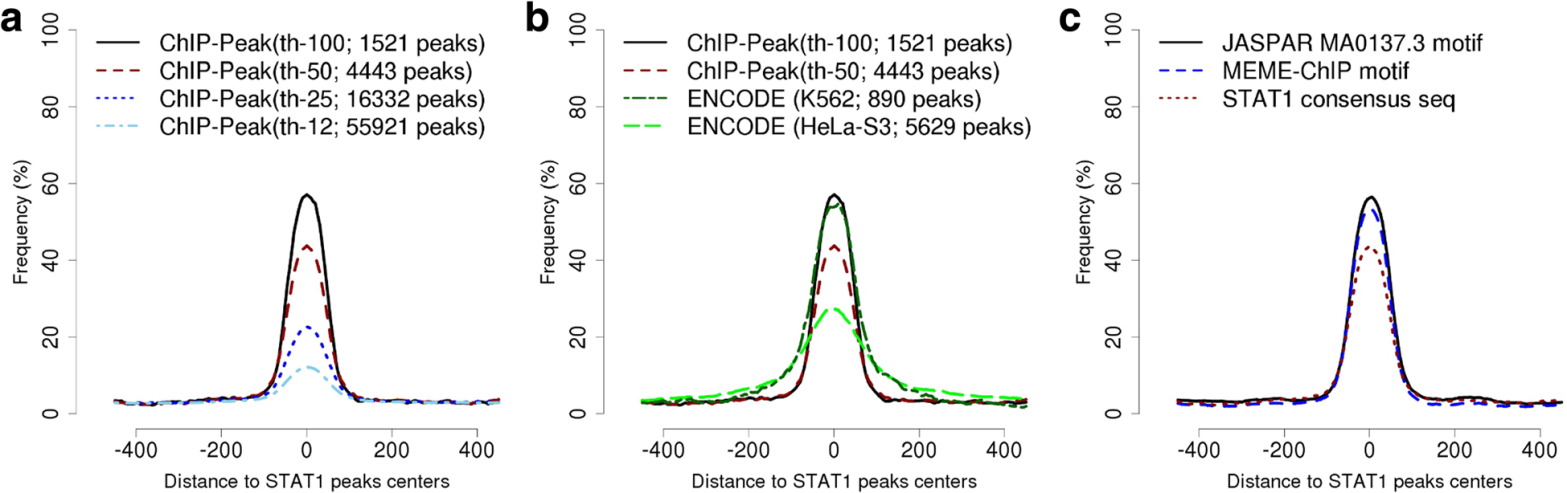
Motif enrichment analysis. **a** STAT1 consensus sequence (TTCNNNGAA) enrichment in peak lists obtained at various tag thresholds. **b** Comparisons of peak lists derived with ChIP-Peak from data published in [45] versus peak lists published by ENCODE. Here, consensus sequence enrichment serves as a proxy for enrichment in true binding sites. Note that a fair comparison is only possible between peak lists of similar size. **c** Comparative evaluation of three alternative STAT1 binding motif descriptions: (i) consensus sequence TTCNNNGAA, (ii) PWM from JASPAR and (iii) MEME-ChIP-derived PWM from the peak regions identified by ChIP-Peak (tag threshold 100)

**Fig. 6:**
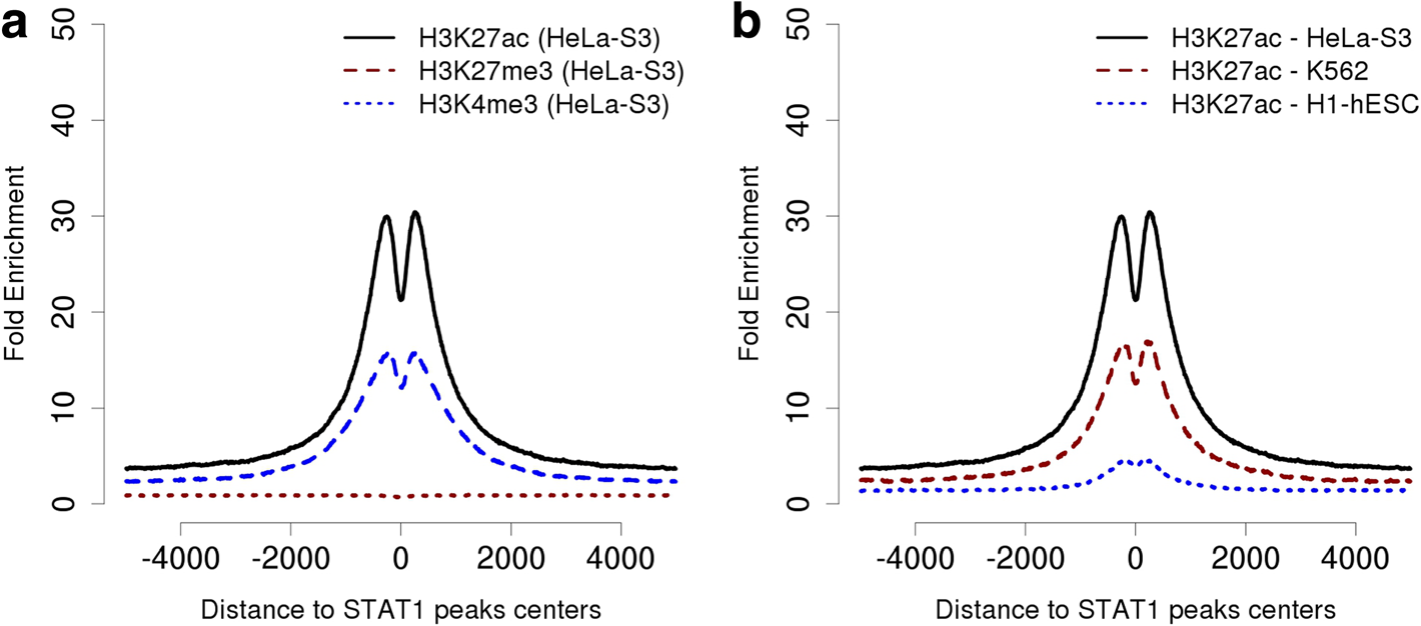
Histone modifications around STAT1 peaks. **a** Distribution of three histone marks around STAT1 peaks from interferon-γ stimulated HeLa cells. Note that the histone marks have been assayed in non-stimulated HeLa cells where STAT1 is not supposed to bind to any of its genomic target sites. **b** H3K27ac marks around STAT1 peaks in HeLa and other cell types

**Fig. 7:**
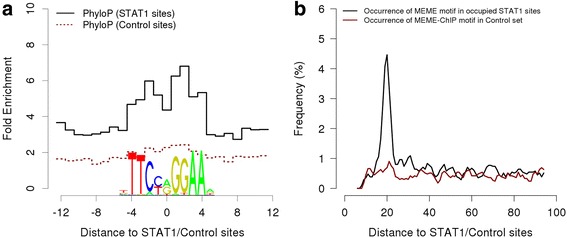
High resolution aggregation plots for in vivo occupied STAT1 sites. **a** Single-base resolution phyloP profile around STAT1 motifs aligned with the sequence Logo of the JASPAR STAT1 matrix. Note the reduced conservation at the weakly conserved central base of the near-palindromic STAT1 motif. **b** Occurrence and distance preference of a second STAT1 motif downstream of an in vivo bound motif. The control set consists of motif matches outside STAT1 peak regions. The MEME-ChIP derived PWM was used for this analysis

Here we will focus on the biological motivation for the different types of analyses and on the interpretation of the results. Detailed step-by-step instructions on how to reproduce the results shown in Figs. 3, 4, 5, 6 and 7 via the web server can be found in Additional file 1: Text S4. Note further that the graphics shown in this paper are not screenshots of the server output pages. They typically combine results from different program runs and were generated by downloading the results in text format and subsequently processing them with the R software.

### 5′-3′ end correlation analysis

We start by generating a 5′-3′ strand correlation plot using ChIP-Cor on the STAT1 data set mentioned above. We use the 5′ (+ strand) tags as reference feature and compute the frequencies of 3′ tags as a function of the distance from the reference feature (Fig. 3a). ChIP-Cor offers several options for scaling the abundance of the target feature. Here, we choose “count density”, which is defined as the number of target feature tags per base pair. We note a Gaussian peak with a maximum at about position +150, suggesting that the average length of an immunoprecipitated fragment is about 150 bp. In all subsequent analyses, we will therefore use half of this value (75 bp) as centering distance for combining the 5′ and 3′ tags from this experiment. Repeating the same analysis with the control sample produces an essentially flat profile (Fig. 3a), consistent with the assumption that virtually all tags from this experiment represent background noise.

Next, we generate a so-called autocorrelation plot for centered STAT1 tags against themselves (same reference and target feature). We see again a Gaussian peak this time with a maximum at 0 (Fig. 3b). The ChIP-Cor server automatically attempts to fit the correlation histogram to a Gaussian curve. If successful, the results of the fit can be accessed via a hyperlink on the output page. Results are provided in graphical and textual form. The text output file contains recommended parameters for the subsequent peak finding step (in Additional file 1: Figure S1).

### Peak detection

The Gaussian fit to the auto-correlation plot suggests a window size of 286 bp and a threshold value of 12 tags for peak finding. We round the window to 300. Running then ChIP-Peak with the recommended parameters returns 55’922 peaks. We have to be aware that 12 is a minimal threshold intended to maximize sensitivity. For many types of downstream analysis more stringently selected peak lists are preferable. We therefore repeat ChIP-Peak with higher thresholds of 25, 50 and 100 tags and obtain 16’332, 4’442 and 1’521 peaks, respectively.

ChIP-Peak returns peak lists in three formats, SGA, FPS, and BED. It is recommended to save them in all three formats for further analysis. Moreover, the output page contains an action button allowing for remapping of the chromosomal coordinates to other genome assemblies.

Some of the identified STAT1 peaks fall into repetitive elements of the human genome. These peaks may cause problems for certain types of downstream analysis, in particular DNA motif discovery. Since all ChIP-Seq server input forms allow users to filter out tags falling into repeat regions, we rerun ChIP-Peak once more with the RepeatMasker checkbox activated.

### Peak analysis with external tools

The output page of ChIP-Peak contains links to other web resources (Fig. 2b). The link to the UCSC Genome Browser enables the user to view individual STAT1 peaks in the context of other genomic features. The hyperlink to the GREAT server serves for GO term enrichment analysis of the genes in the neighborhood of peaks (Fig. 4a). We note that the majority of terms relate to cytokine mediated signaling consistent with the reported biological function of STAT1.

Another topic of interest is the location of TF binding peaks relative to protein coding genes. One web-based resource performing such an analysis is Nebula. It returns graphics showing the abundance of peaks within promoter regions, gene bodies, intergenic regions, and components of genes (Fig. 4b). Nebula also returns a peak annotation table, indicating for each peak the nearest gene and its relative location to that gene.

### Motif studies in peak regions

STAT1 is known to bind to a DNA motif approximately described by the consensus sequence TTCNNNGAA. If the peaks found by ChIP-Peak are indeed real binding sites, one would expect this motif to be over-represented near the peak center positions. In fact, motif enrichment analysis is commonly used for benchmarking the performance of ChIP-seq peak finders [47]. The OProf program of the SSA server can be used for this purpose. It returns a graph showing the percentage of sequences containing a motif in a sliding window along genomic sequences aligned on a reference position, in this case the peak center. Figure 5a shows the motif occurrence profiles for TTCNNNGAA for the four different STAT1 peak lists obtained with different tag thresholds. With all peak lists, we see a clear enrichment of STAT1 motifs near position zero (the reported peak center). As expected, the peak height is inversely correlated to the number of peaks. Note however that in absolute terms, the number of motif-containing peaks is highest in the peak list obtained at the lowest tag threshold 12: 13.7% of 55,921 = 7661 as compared to 25.1% of 16,332 = 4099 for tag threshold 25.

The OProf server provides access to a large number of ChIP-seq peak lists, including two STAT1 peak lists from the ENCODE consortium. Figure 5b shows the consensus sequence enrichment profiles for the ENCODE peak lists together with the two high-threshold peak lists generated with the ChIP-Seq server. We note that our peak lists compare favorably to ENCODE peak lists with similar peak numbers, both in terms of enrichment (peak height) and positional resolution (peak width).

For most TFs, a consensus sequence can only provide an approximation of the true binding motif. Position weight matrices (PWMs) are generally considered superior tools for describing the binding specificity. The OProf server provides menu-driven access to PWMs from several public resources, including a STAT1 matrix from the JASPAR database [48]. We may wonder whether an even better matrix could be obtained by applying a *de novo* motif discovery program to ChIP-seq peak regions. To test this, we extract sequences from position −60 to +60 relative to the peak center positions from the repeat-masked peak list obtained with tag threshold 100. The sequences can be transferred via copy-paste to the MEME-ChIP server. Since we expect the STAT1 binding motif to be palindromic, we restrict the search to palindromic motifs. The resulting PWM is shown in Additional file 1: Text S4.

Figure 5c shows motif enrichment profiles for the STAT1 consensus sequence, the JASPAR matrix, and the *de novo* generated matrix. All motifs were searched at an equal random discovery rate, which is a condition for fair comparison. Among the two PWM-based motifs, we note a slightly better performance of the JASPAR matrix suggesting that this PWM is near-optimal.

### Exploring the genomic context of STAT1 peaks

ChIP-Cor enables the user to generate aggregation plots (APs) for peak lists with a great variety of target features. We first investigate whether the STAT1 binding sites are associated with active or repressive histone marks. Since the STAT1 binding experiment was carried out in HeLa cells, we choose histone modification data from the same cell type generated by the ENCODE consortium. Specifically, we are going to test an active promoter mark (H3K4me3), an active enhancer mark (H3K27ac) and a repressive chromatin mark (H3K27me3). Remember in this context, that STAT1 peaks were discovered in HeLa cells that were stimulated with interferon-γ. On the other hand, the histone modification maps from ENCODE were obtained from non-stimulated cells, in which STAT1 is not supposed to bind to any genomic target sites. The results are shown in Fig. 6a. We see that STAT1 peaks fall into regions of about 500 base-pairs which are 15-fold enriched in H3K27ac and 7-fold in H3K4me3 compared to the background level. Conversely, no enrichment is seen for H3K27me3 in the vicinity of STAT1 peaks. These results suggest that STAT1 binds primarily to regions that are already in an active chromatin state before interferon-γ induction. Note further the bimodal distribution of the active histone marks with maxima symmetrically positioned on either side of the peak center. This may indicate that STAT1 preferentially binds to target sites that are nucleosome-free in unstimulated cells.

We may wonder whether genomic regions bound by STAT1 in HeLa cells are also in an active state and nucleosome-free in other cell types. To answer this question, we generate APs for H3K27ac in the embryonic stem cell line H1-hESC and the leukemia-derived cell line K562 (Fig. 6b). We see an approximately two-fold higher enrichment in HeLa cells over K562 and an almost flat H3K27ac profile in H1-hESC, suggesting a substantial degree of tissue-specificity of the regulatory regions that are *bona fide* accessible to STAT1 by virtue of their chromatin state.

In addition, we may explore DNase I hypersensitivity, sequence conservation, and population variation data near STAT1 sites (in Additional file 1: Figure S2). The results of such an analysis can be summarized as follows: STAT1 peaks occur preferentially within DNase hypersensitive regions of up to 500 bp. Increased cross-species conservation is observed in a slightly narrower region of about 300 bp. Consistent with this finding, we see depletion of indel variation in the same region. However, contrary to expectation, there appears to be no depletion of common SNPs.

### High resolution aggregation plots for bound PWM matches

According to the motif occurrence analysis (Fig. 5), our peak lists have a positional precision of ±50 bp. Aggregation plots of potentially higher resolution could be obtained by using the STAT1 motifs found in peak regions as anchor points. We use the SSA program FindM to generate a genomic coordinate list of STAT1 motifs (JASPAR PWM matches) that occur within 75 bp from the STAT1 peak center position. To generate a random control set, we also collect PWM matches from genomic regions far downstream of the peak regions (+10000 to +12000 relative to the peak center). The size of the control regions has been chosen such as to generate a STAT1 motif list of approximately the same size.

Figure 7a shows single base resolution plots for sequence conservation using PhyloP scores from the UCSC Genome Browser database. Also included in the figure is the sequence logo of the STAT1 matrix aligned with the motif location on the horizontal axis. We see increased sequence conservation within the 9 bp regions that make up the STAT1 core motif. As expected, the center position, which is essentially unconstrained, is not more conserved than the flanking regions. Note further that the random control sites, most of which are presumably not bound by STAT1, show a much lower degree of sequence conservation which furthermore does not correlate with the column heights in the sequence logo.

Lists of exact motif coordinates rather than fuzzy peak center positions are also useful to investigate interactions between sequence motifs. Here we ask the question whether in vivo STAT1 binding motifs preferentially occur as pairs separated by a characteristic distance from each other. Figure 7b shows a STAT1 motif autocorrelation plot, i.e. a single-base resolution occurrence profile of STAT1 PWM matches downstream of in vivo occupied STAT1 motifs. We see a narrow peak (±2 bp) centered 21 bp downstream of the in vivo bound motifs which is absent in a plot generated with the control set. This previously observed preferential occurrence of STAT1 binding site pairs at a center-to-center distance of two helical turns [22] could be explained by a tetrameric binding mode experimentally documented for some members of the STAT1 family.

### Comparison of the ChIP-seq server with other resources

A comprehensive survey of all currently available software resources for ChIP-seq data would be beyond the scope of this article. We therefore deliberately restrict our comparison to similar resources meeting two criteria: (i) being available over a web interface (ii) and including applications that accept a read alignment file in BED or BAM format as input. This excludes software packages which require a local installation (*see* Background Section), web-based resources supporting only downstream analysis after peak finding, e.g. EpiExplorer [49] or ChIPseek [50]. A comparison of the features and services offered by the remaining resources is given in Additional file 1: Table S3.

The Galaxy-based Cistrome platform comes perhaps the closest to our resources. Like the ChIP-Seq server, it provides access to a large server-resident collection of public data. For transcription factors, ChIP-seq data are only offered as peak lists, not as files containing the coordinates of mapped sequence tags. Cistrome offers additional statistical analysis tools and further supports RNA-seq data analysis. The other three Galaxy servers, Galaxy main [51], Nebula and Galaxeast offer read mapping as an additional service and thus enable users to start with raw sequence files. Nebula is our preferred resource for peak annotation. In addition, it serves as a web interface for a number of more advanced in-house developed programs by the Nebula team.

Among the non-Galaxy based resources, GeneProf [52] and HTStation [53] offer the most comprehensive ChIP-seq data analysis services. GeneProf provides access to a large database of experiments and precomputed results. Workflows are displayed through an intuitive graphical interface enabling users to download the input files, intermediate data and final results via clickable icons. HTStation offers a completely automatized ChIP-seq data analysis pipeline in batch mode, including quality control, peak finding and DNA motif discovery with MEME-ChIP. ColoWeb [54] is a more specialized resource primarily designed to make APs with server-resident histone modification data and TSSs or ChIP-seq peaks as anchor points. W-ChIPeaks [55] is essentially a web-based peak finder.

Compared to the ChIP-Seq web server design, the Galaxy platform offers a number of generally useful functionalities such as the possibility to store private data on the server side, and to save workflows for later use with new data. Consistent with the guiding principles of the ChIP-Seq command line tools, we have chosen a lighter design. The main goal is to offer users the opportunity to explore a large number of public data sets rapidly and in a highly interactive manner. The direct navigation buttons, which connect web server output pages to input forms, allow users to carry out complex analysis tasks with a minimal number of mouse clicks. For instance, after importing an alignment file in BAM or BED format via the ChIP-Convert page, it takes less than one minute (and only three mouse clicks) to extract peaks and to make a motif enrichment plot of the kind shown in Fig. 5.

## Conclusions

We have presented in some detail the ChIP-Seq web server, the command-line programs behind the server, and the data back-end called MGA repository. The three resources are designed to function together. Nevertheless, the command-line tools and MGA repository can be viewed as independent resources distributed via different channels and potentially useful to different researchers. The command line tools may fill gaps in the software repertoire of a computational genomics group. The MGA repository is to our knowledge the largest ChIP-seq data collection made available in a completely standardized format, and for this reason may also be appreciated by computational biologists. The web server is probably appealing to a more diverse user community, ranging from bench biologists primarily interested in analyzing their own data, to pure *in silico* biologists investigating the principles of gene regulation by exploring large public data sets.

By providing access to a rich public data collection, the ChIP-Seq server is also ideally suited as an educational tool. Teachers can illustrate technical characteristics of ChIP-seq data as well as biological phenomena revealed by this technology with a great variety of real data examples. Students and prospective users of ChIP-seq assays have the opportunity to get a feeling for the data and learn about analysis methods by re-analyzing data and reproducing results from landmark papers.

Unlike most other resources, the ChIP-Seq server is an open system designed to be used in conjunction with other web servers or locally installed software packages. The creation of a comprehensive public data analysis platform has never been our objective. Neither will it be our policy in the future to add functionalities that are offered elsewhere. Rather we will continue to promote interoperability with other resources by supporting new data exchange formats as they become available. Support of reproducible computational research is another guiding principle of our development efforts. We will take any measures to make sure that the methods behind our web servers are transparent and that any result returned can be reproduced from the Unix command line with data files that can be downloaded from the MGA repository and publicly available open source software.

The extension and curation of the public data collection at the back-end of the server will be a high priority in future development efforts. While it is clear that we will not have the resources to comprehensively mirror all future public data sets, we will try to offer a balanced mixture of data sets from high impact papers and large consortium efforts responding to the demand of a diverse user community. Specifically, we plan to expand the hitherto somewhat neglected data collections for invertebrate and plant species. As a second priority, we intend to enhance the usability of our resources by organizing hands-on courses for prospective users at least once a year, and by extending the collection of tutorials and other E-learning tools posted on our website. Briefly, our mission continues to be making ChIP-seq data more usable and more widely used.

## Additional file

Additional file 1: Supplementary Material: **Texts S1–S4**, **Figures S1–S2**, **Tables S1**–**S3**. (PDF 1043 kb)

## Abbreviations

AP: Aggregation plot
ChIP: Chromatin immuno-precipitation
ChIP-seq: ChIP combined with high-throughput sequencing
FPS: Functional position set (name of the file format used by the SSA package)
GO: Gene ontology
MGA: Mass sequences of genome annotation
NGS: Next-generation sequencing
PWM: Position weight matrix
SSA: Signal search analysis (name if a DNA motif analysis package)
TF: Transcription factor
TSS: Transcription start site
