# Supplementary material for: The ChIP-Seq tools and web server: a resource for analyzing ChIP-seq and other types of genomic data
Source: BMC Genomics. 2016 Nov 18;17:938. doi: 10.1186/s12864-016-3288-8 (PMC5116162; doi:10.1186/s12864-016-3288-8)

Supplementary Material

The ChIP-Seq tools and web server: a resource for analyzing ChIP-seq and other types of mass genome annotation data

Giovanna Ambrosini, René Dreos, Sunil Kumar, Philipp Bucher

Contents:

Text S1. Gaussian curve fitting

Text S2. Data format and conversion

Text S3. Pseudo-code for main algorithms

Text S4. Step-by-step instruction for reproducing results

Table S1. ChIP-seq command line programs

Table S2. Contents of the MGA repository

Table S3. Survey of related resources

Figure S1. Peak fitting parameters (from ChIP-Cor)

Figure S2. DNAse I hypersensitivity, sequence conservation and population variation near STAT1 sites

Text S1. Gaussian curve fitting

To best fit the correlation data around the main peak, if it exists, we use the Gaussian distribution given by the following formula:

$$a + \frac{b}{\sqrt{2\pi\sigma^2}} e^{-(x-\mu)^2/2\sigma^2}$$

where μ is the mean of the distribution and represents the peak location and σ is the standard deviation. The parameter *b* reflects the peak volume, whereas the parameter *a* shifts the curve along the y axis (and is related to the expected number of background counts under the peak area ($2*\sigma*a$) – *a* ~ background density). The goodness-of-fit is summarized by the value of the "residual" given as a result of the fitting function. In a typical peak finding application, one would first guess the centering distance by the 5'-3' end correlation: μ can be used as an estimate of the average fragment size.

Then one would use ChIP-Cor again for an autocorrelation analysis with centered ChIP-Seq tags to estimate the parameters for peak finding: the average peak width or window size, and the peak threshold or count threshold. The peak width is computed as twice the σ. For computing the peak threshold, we assume that the counts in a window are Poisson distributed. We first determine the low and high peak thresholds using the R Poisson function which returns counts for given quantiles. We then compute the expected number of peaks for all peak thresholds within the interesting range. These values are displayed in the ChIP-Cor output page. Results for STAT1 (Robertson et al., 2007) are listed below:

Peak Finding Parameters Estimate

Av. BG density (cnts)	0.0109
Peak window width (bp)	253
Peak threshold (cnts)	12

**Peak Threshold (cnts)**	**Expected Random Peaks**
8	27718
9	7440
10	1825
11	412
12	86

For peak fitting we recommend using a minimal window size of 10 (histogram bin size) in ChIP-Cor.

Given the results above, we can reasonably choose a peak count threshold (*t*) of 15 as a significant enrichment level threshold, and a peak width (*w*) of 200 (bp). Our peak recognition algorithm is based on the assumption of having a uniform background fragments distribution throughout the genome.

Alternatively, the peak threshold can be expressed as enrichment factor relative to the background fragment distribution. The web server suggests a relative enrichment factor of 10 that works well in most cases.

Text S2. Data format and conversion

We also provide a series of auxiliary tools (most of them are Perl scripts) that can be used to perform format conversion tasks.

Most of the ChIP-seq data sets come in BAM or BED formats. If you have BAM files, and you need to convert them to SGA format, the steps to follow are:

- Find out which genome assembly has been used to generate the BAM files, and make sure that the chromosome names agree with the naming scheme of the UCSC genome browser.
- Then use the following type of command:

bamToBed -i reads.bam | bed2sga.pl -f <feature> -s <species> | sort -s -k1,1 -k3,3n -k4,4 | compactsga > reads.sga

The program *bamToBed* belongs to the **BEDTools** package, a suite of utilities for comparing genomic features that can be installed from:

https://github.com/arq5x/bedtools2

bamToBed converts BAM alignments to BED format. The '*bed2sga.pl*' tool is a perl script that converts BED files to SGA format. To find instructions how to use it, type:

bed2sga.pl -h

SGA format requires a *feature* field (the second field) containing a character string that defines the genomic feature represented by the file. The BED file does not have an equivalent field. You therefore need to supply a feature for the conversion via the *-f* option. The *species* is the name used by UCSC for the genome assembly to which the reads have been mapped (*e.g.* hg18, hg19, mm9, dm3, etc.). *bed2sga.pl* will automatically convert UCSC chromosome names into corresponding NCBI/RefSeq accession numbers. Currently we provide chromosome ID conversion for the following assemblies: hg19, *hg18*, *mm8*, *mm9*, mm10, *ce6*, *dm3*, dm6, *danRer4*, *danRer5*, danRer7, *rn4*, rn5, *panTro2*, *sacCer3, ararTha1, and spo2 (S.pombe)*. The *species* is only required if one wants to translate UCSC-based chromosome names into NCBI RefSeq identifiers for comparison with data that use NCBI identifiers.

The information required to map NCBI RefSeq identifiers into chromosome numbers is provided by a binary table called *chro_idx.nstorage*. The binary file *chro_idx.nstorage* includes a hash table in Perl that, for each assembly, stores chromosome number-NCBI identifier pairs as well as chromosome lengths indexed by chromosome NCBI identifiers.

The reasons why sorting is required have been explained before. The program '*compactsga*' is a C program, which merges lines corresponding to the same genome position (identical sequence name, position and strand). For instance, the two input lines:

```
NC_000001.9        H3K4me3    5011    +    1
NC_000001.9        H3K4me3    5011    +    1
```

would be replaced by the following single line:

```
NC_000001.9        H3K4me3    5011    +    2
```

Compacting sga files saves space and reduces program execution time, but unlike sorting is not formally required by the ChIP-Seq tools.

The *bed2sga.pl* has two basic modes of operations, *centered* and *oriented*. In the *centered* mode, the midpoint between the *start* and *end* position from the BED line (2nd and 3rd field) will be used as position. In the *oriented* mode, the conversion depends on the *strand* indicated in the BED file (6th

field). If the strand is + then the value of the *start* field will be incremented by one and used as position in the SGA file. If the strand is −, the value of the *end* field will be used as position in the SGA file. Incrementing the start position in *oriented* mode is necessary because the BED format has a "zero-based" numbering system for chromosomal regions whereas SGA has a 1-based numbering system. The behaviour of the two conversion modes is illustrated by the following example:

BED input:

```
chr1    100000266   100000291    .  0   +
chr1    100000383   100000408    .  0   -
```

SGA output, centered mode:

```
NC_000001.9   CHIPSEQ   100000278   +   1
NC_000001.9   CHIPSEQ   100000395   -   1
```

SGA output, oriented mode:

```
NC_000001.9   CHIPSEQ   100000267   +   1
NC_000001.9   CHIPSEQ   100000408   -   1
```

By the default, the conversion mode depends on the contents of the BED file. If the strand field (which is optional in BED) is present, the conversion will be done in *oriented* mode. Otherwise, the conversion will be done in centered mode, and the strand field will be set to zero. However, centered mode can be forced by the command line option *–c*.

The most common command pipeline to perform BED-to-SGA conversion is the following:

bed2sga.pl -f <feature> -s <species> reads.bed | sort -s -k1,1 -k3,3n -k4,4 | compactsga > reads.sga

There are several additional reformatting programs that may be useful in certain situations.

The formatting programs are typically used together with other formatting tools belonging to publicly available software packages.

One example of such software is the liftOver program from UCSC that can be used to convert chromosomal coordinates in a BED file from one genome assembly to another, *e.g.*:

liftOver input.bed /db/liftOver/mm8ToMm9.over.chain.gz output.bed trash

Note that liftOver chain files can be downloaded from the UCSC site.

Additional conversion scripts include:

sga2bed.pl	SGA to BED
partit2bed.pl	Output of Partitioning tool (special SGA) to BED
fps2sga.pl	FPS to SGA
sga2fps.pl	SGA to FPS
gff2sga.pl	GFF to SGA
sga2gff.pl	SGA to GFF
sga2wigFS.pl	SGA to WIG (wiggle) fixedStep format
sga2wigVS.pl	SGA to WIG variableStep format

The last two programs produce custom track files that can be uploaded to the UCSC genome browser for visualization. The FPS (Functional Position Set) format is the specific format used by the Signal Search Analysis tools (http://seqanswers.com/wiki/FPS).

Text S3. Pseudo-code for main algorithms

ChIP-Cor basic algorithm

```
Initialization:
   /* Input parameters: */
       character ref_name, ref_strand, tar_name, tar_strand;
       intger start_pos, end_pos, window_size;
       integer L ← (end_pos – start_pos) / window_size;
       vector float histogram(0) to histogram(L-1) ← 0;
       integer i ← 0  /* reference feature index */, j ← 0  /*target feature index */;
       vector integer ref.pos(),ref.counts(), tar.pos(), tar.counts(), target.ptr();
Sequential reading of SGA file:
   Until End of File (EOF) do:
         /* Read SGA line */
          Get seq_name,  feature, pos, strand, counts
          If seq_name  is new do:
                I ← i; J ← j; updateHistogram(histogram, I, J);  I ← 0; J ←0;
          End If
          If (feature = 'ref_name' and strand = 'ref_strand') do: /* Process Reference Feature */
                If (pos = 'ref.pos(i)') do:  ref.counts(i) ←  ref.counts(i) + counts;
                Else do: i++; ref.pos(i) ←pos;  ref.counts(i) ← counts; target.ptr(i ) ← j;  End If
          Else If (feature = 'tar_name' and strand = 'tar_strand') do: /* Process Target Feature */
                If (pos = 'trg.pos(j)') do : trg.counts(j) ← trg.counts(j) + counts;
                Else do:  j++; ref.pos(j) ← pos, ref.counts(j) ←counts;  End If
          End If
    End loop
Function updateHistogram(histogram, I, J): void
Begin
   For i ← 1 to I by 1 do:
         j = target.ptr(i);
         While (j > 0 and (tar.pos(j)  − pos) ≤ start_pos) do:  #Examine upstream target positions
                k ← int( (tar.pos(j) – ref.pos(i) – start_pos) / window_size );
               histogram(k) ← histogram(k) + ref.counts(i) × trg.counts(j);
               j--;
         End loop
         j = target.ptr(i) + 1;
         While (j ≤ J and (tar.pos(j) – pos)  ≤ start_pos) do: #Examine downstream target positions
               k ← int( (tar.pos(j) – ref.pos(i) – start_pos) / window_size );
               histogram(k) ← histogram(k) + ref.counts(i) × trg.counts(j);
               j++;
         End loop
   End loop
End
```

ChIP-Peak basic algorithm

```
Initialization:
  /* Input parameters: */
     character feature_name;
     integer peak_threshold, window_size, vicinity_range;
     integer i ← 0;  /* feature index */
     character seq_id;
     vector character feature.name();
     vector integer feature.pos(), feature.counts(), peak.pos(), peak.counts();
Sequential reading of SGA file:
  Until End of File (EOF) do:
       /* Read SGA line */
       Get seq_name, feature, pos, strand, counts
       If seq_name is new do:
            seq_id ← seq_name;
            feature.pos(0) ← feature.pos(1) – window_size/2 -1;
            feature.pos(i + 1) ← feature.pos(i) + window_size/2 +1;
            locatePeaks(i), i ← 0;
       End If
       If (feature = 'feature_name') do:
            If (pos = 'feature.pos(i)') do: feature.counts(i) ← feature.counts(i) + counts;
            Else do: i ++, feature.pos(i) ← pos, feature.counts(i) ← counts; End If
       End If
  End loop
Function locatePeaks(I): void
Begin
  integer j ← 0;  /* peak index */
  For i ← 1 to I by 1 do:
       integer sum ← feature.counts(i);
       k ← i – 1;
       While k > 0 and (feature.pos(k) >= feature.pos(i) - window_size/2) do:
            sum ← sum + feature.counts(k);
            k--;
       End loop
       k ← i + 1;
       While k < 'I' and (feature.pos(k) <= 'feature.pos(i) + window_size/2') do:
            sum ← sum + feature.counts(k);
            k++;
       End loop
       If (sum >= peak_threshold ) do: /* This is a peak because sum of counts is above threshold */
            peak.pos(j) ← feature.pos(i);
            peak.counts(j) ← sum;
            feature.ptr(j) ← i;
```

```
            j++;
        End If
    End loop
    /* Keep only peaks that are local maxima within a vicinity range of ±vicinity_range  (lm(i) = 3) */
    integer max = 1;
    i ← 2;
    While (i <= 'j') do:   /* Select maxima within forward path  */
        If (peak.pos(i) > 'peak.pos(max) + vicinity_range') do:
            lm(max) ← lm(max) + 1;
            max ← i;
        Else If (peak.counts(i) >  'peak.counts(max)') do:
            max ← i;
        End If
        i++;
    End loop
    lm(max) ← lm(max) + 1;
    max ← j;
    i ← j – 1;
    While (i >= '0') do:  /* Select maxima within backward path */
        If (peak.pos(i) <' peak.pos(max) - vicinity_range')  do:
            lm(max) ← lm + 2;
            max ← i;
        Else If (peak.counts(i) > 'peak.counts(max)') do:
            max ← i;
        End If
        i--;
    End loop
    lm(max) ← lm(max) + 2;
    /* Print out local maxima positions (lm(i) = '3') */
    For i ← 1 to j by 1 do:
        If (lm(i) = '3') do:
            print (seq_id, feature.name(i), peak.pos(i), peak.counts(i));
        End If
    End loop
End
```

ChIP-Part basic algorithm

```
Initialization:
    /* Input parameters: */
        character feature_name;
        integer threshold, penalty;
        integer i ← 0, last_position;
```

```
    character seq_id;
    vector character feature.name();
    vector integer feature.pos(), feature.counts();
Sequential reading of SGA file:
  Until End of File (EOF) do:
      /* Read SGA line */
      Get seq_name, feature, pos, strand, counts
      If seq_name is new do:
          seq_id ← seq_name;
          feature.pos(i + 1) ← last_position;  feature_counts(i + 1) ← 0;
          splitSeq(i, last_position), i ← 0;
      End If
      If feature = 'feature_name' do:
          If pos = 'feature.pos(i)': feature.counts(i) ← feature.counts(i) + counts;
          Else do: i ← i + 1, feature.pos(i) ← pos, feature.counts(i) ← counts; End If
          last_position ← feature.pos(i);
      End If
  End loop
Function splitSeq(I, last_position): void
Begin
  /* The Partitioning Algorithm splits the sequence in regions so as to maximize the following scoring
function, for each position i:
      - vector float X  is the score when partitioning ends in signal-enriched states
      - vector float Y is the score when partitioning ends in signal-depleted states
      - integer q(i) is the trace-back code for all possible transitions:
          - signal-poor to signal-rich (YX)
          - extend signal-rich   (XX)
          - signal-rich to signal-poor to signal-rich (ZX)
          - signal-rich to signal-poor (XY)
          - extend signal-poor  (YY)
  */
  float Xmax ← 0, Ymax ← 0;
  float XX, YX, ZX, YY, XY;
  matrix integer Reg();
  vector integer counts();
  For i ←1 to I + 1 by 1 do:
    /* Compute (genome-wide) Scoring Function: */
    XX ← Xmax + feature.counts(i) – (feature.pos(i) - feature.pos(i-1))*Threshold;
    YX ← Ymax + Penalty + feature.counts(i)–Threshold + (feature.pos(i)-feature.pos(i-1) -1)*Threshold;
    ZX ← Xmax +2*Penalty +feature.counts(i)–Threshold + (feature.pos(i)-feature.pos(i-1) -1)*Threshold;
    YY ← Ymax + (feature.pos(i) - feature.pos(i-1)) * Threshold - feature.counts(i);
    XY ← Xmax + Penalty + (feature.pos(i) - feature.pos(i-1))*Threshold - feature.counts(i);
    vector float X ← vector(XX, YX, ZX);
    vector float Y ← vector(YY, XY);
```

```
    Xmax ← max(X);
    Ymax ← max(Y);
    /* Fill in trace-back codes: */
       q(i) ← 0   if  Xmax = 'XX' and  Ymax = 'YY';  /* extend signal-rich region */
            ← 1   if  Xmax = 'ZX' and  Ymax = 'YY';  /* enter signal-rich region */
            ← 2   if  Xmax = 'YX' and  Ymax = 'YY';  /* signal-rich to signal-poor region */
            ← 3   if  Xmax = 'XX' and  Ymax = 'XY';  /* extend signal-rich region or enter signal-rich */
            ← 4   if  Xmax = 'ZX' and  Ymax = 'XY';  /* enter signal-rich region */
            ← 5   if  Xmax = 'YX' and  Ymax = 'XY';  /* enter signal-rich or end signal-rich region */
  End loop
  /* Trace-back to identify the signal-rich regions:  Reg(0, k), Reg(1, k) , counts[k] */
  integer k ← 1;
  If (Xmax > 'Ymax') do:
     Reg(0, k) ← 0;  Reg(1, k) ← last_position;
  Else do
     Reg(0, k) = 0;  Reg(1, k) = 0
  End If
  For i ← I + 1 to 1 by -1 do:
       If  Reg(1, k) = '0' do:
            If  q(i) > '2' do:  Reg(1, k) ← feature.pos(i -1)  /* Enter a signal-rich region
                                                                  from a signal-poor region*/
       Else do:
            If  q(i) > '2'  do:  q(i) = q(i) - 3;  End if
            If  q(i) = '1'  do:    /* Enter signal-rich region for the first time, close the previous one */
                   Reg(0, k) ←  feature.pos(i); counts(k) ← counts(k) + feature.counts(i);
                   k++;
                   Reg(0, k) ← 0;   Reg(1, k) ← feature.pos(i -1);
            Else If  q(i) = '2'  do :  /* End of signal-rich region, close it. Next is signal-poor region */
                   Reg(0, k) ← feature.pos(i); counts(k) ← counts(k) + feature.counts(i);
                   k ++;
                   Reg(0, k) ← 0;   Reg(1, k) ← 0;
             Else  do:     /* Extend signal-rich region  */
                   counts(k) ← counts(k) + feature.counts(i);
             End If
       End If
  End loop
  /* Print out signal-rich regions  */
  For i ← k to 1 by -1  do:
     print (seq_id, feature.name(i), Reg(0, i), '+', counts(i));  /* Print 'start' position of each region */
     print (seq_id, eature.name(i), Reg(1, i), '-', counts(i));    /* Print 'end' position of each region */
  End loop
End
```

Text S4. Step-by-step instructions for reproducing results.

This Section presents computational recipes for reproducing all Figures of this paper which show results generated with the ChIP-Seq and SSA-servers (Figures 3-7 and S2). The first part provides step-by-step instructions for generating the results via the web interface. The second part shows the R code that has been used to generate the Figures. Please note that the R code will only work if all results have been saved under the names suggested in the first part.

For a more detailed description of how to use the ChIP-Seq and SSA analysis tools, please refer to the on-line basic tutorial at:

http://ccg.vital-it.ch/chipseq/doc/chipseq_tutorial.pdf

Figure 3. 5'-3'end correlation and autocorrelation plots. (a) 5'-3' end correlation plot for STAT1 ChIP-seq tags from interferon-γ stimulated and unstimulated HeLa cells. The horizontal position of the peak maximum suggests an average fragment size of about 150 bp. **(b)** Autocorrelation plot of 75bp-centered STAT1 ChIP-seq tags from stimulated cells.

Open the ChIP-Cor server input page at:

http://ccg.vital-it.ch/chipseq/chip_cor.php

Fill out the form as shown in Table 1, and click on the Submit button.

ChIP-Seq Input Data Reference Feature	ChIP-Seq Input Data Target Feature
**Select available Data Sets**<br>Genome: H. sapiens (Feb 2009/hg19)<br>Data type: ChIP-seq<br>Series: Robertson 2007 …<br>Sample: HeLa S3 STAT1 stim	**Select available Data Sets**<br>Genome: H. sapiens (Feb 2009/hg19)<br>Data type: ChIP-seq<br>Series: Robertson 2007 …<br>Sample: HeLa S3 STAT1 stim
**Additional Input Data Options**<br>Strand: +<br>Analysis Parameters<br>Range: -1000 to 1000	**Additional Input Data Options**<br>Strand: -
**Histogram Parameters**<br>Window width: 10<br>Count Cut-off: 1<br>Normalization: count density	

Table 1. 5'-3'end correlation with ChIP-Cor.

By clicking on the link "TEXT" save the results as a text file named:

robertson_stat1_correlation.out

Now generate the same plot for the negative control sample:

Hela S3 STAT1 unstim

and save the results as:

robertson_stat1_correlation_unstim.out

To generate the auto-correlation plot displayed in Figure 1b, fill out the ChIP-Cor input form as shown in Table 2 and save the results as:

robertson_stat1_autocorrelation.out

ChIP-Seq Input Data Reference Feature	ChIP-Seq Input Data Target Feature
**Select available Data Sets**	**Select available Data Sets**
Genome: H. sapiens (Feb 2009/hg19)<br>Data type: ChIP-seq<br>Series: Robertson 2007<br>Sample: HeLa S3 STAT1 stim	Genome: H. sapiens (Feb 2009/hg19)<br>Data type: ChIP-seq<br>Series: Robertson 2007<br>Sample: HeLa S3 STAT1 stim
**Additional Input Data Options**	**Additional Input Data Options**
Strand: any<br>Centering: 75	Strand: any<br>Centering: 75
**Analysis Parameters**	
Range: -1000 to 1000	
**Histogram Parameters**	
Window width: 10<br>Count Cut-off: 1<br>Normalization: count density	

Table 2. Autocorrelation with ChIP-Cor.

R Code to generate the Figure

```
-----------------------------------------------------------------------------------------------------------------------------------

stat1.stim.cor <- read.table("robertson_stat1_correlation.out")
stat1.unstim.cor <- read.table("robertson_stat1_correlation_unstim.out")
stat1.stim.auto <- read.table("robertson_stat1_autocorrelation.out")

jpeg("fig1_STAT1_corr_plots.jpg", width = 2000, height = 1000, quality = 100, pointsize=30)
par(mfrow=c(1,2))
plot(stat1.stim.cor, type="l", frame.plot=F, ylim=c(0,0.02), xlab="Distance between 5' and 3' tags", ylab="Count Density", main="", lwd=4, xlim=c(-1000,1000), xaxt="n", cex.axis=1.2, cex.lab=1.2)
axis(1, at=c(-1000,-500,0,150,500,1000), cex.axis=1.2)
text(x=-1050, y=0.022, labels=expression(paste(bold("(a)"))), xpd=NA, cex=2)
points(stat1.unstim.cor, type="l", lty=2, lwd=4, col="darkred")
abline(v=150, lty=3, lwd=2, col="gray")
legend("topleft", legend=c("Hela STAT1 stim","Hela STAT1 unstim"), lty=c(1,2), col=c("black","darkred"), bty="n", lwd=4)
plot(stat1.stim.auto, type="l", frame.plot=F, ylim=c(0,0.04), xlab="Tag relative distance", ylab="Count Density", main="", lwd=4, xlim=c(-1000,1000), cex.axis=1.2, cex.lab=1.2)
text(x=-1050, y=0.044, labels=expression(paste(bold("(b)"))), xpd=NA, cex=2)
abline(v=0, lty=3, lwd=2, col="gray")
legend("topleft", legend=c("Hela STAT1 stim"), lty=c(1), col=c("black"), bty="n", lwd=4)
dev.off()

-----------------------------------------------------------------------------------------------------------------------------------
```

Figure 4. Peak annotation with external tools. (a) GO term enrichment analysis with GREAT. **(b)** Peak location statistics with Nebula.

The output page of ChIP-Peak contains links to several web resources, including a hyperlink to the GREAT server (McLean, et al., 2010), which performs GO enrichment term analysis of the genes in the neighborhood of the peaks. A mouse-click on this link returns the GO term list ('GO Biological Process') corresponding to the STAT1 peak list generated by ChIP-Peak. To visualize the GO term table as a bar chart, select 'Bar chart of current sorted value' under the 'Visualize this table' menu. The result is shown in Figure 5a. The direct link to GREAT is only provided for peak lists that include less than 20'000 peaks.

For peak annotation (Figure 5b) we propose to use Nebula (Boeva, et al., 2012) and proceed as follows.

We first run ChIP-Peak with threshold 50 (tag counts) and Repeat Masker on. The peak lists are posted in three formats, SGA, FPS, and BED. For Nebula, we save the peak list in BED format (by right-clicking on the link 'BED File') under the name:

stat1_t50_rmsk_hg19.out

Go to Nebula and follow the instructions given below:

- Import the BED-formatted peak file generated with ChIP-Peak by selecting 'Upload File' under 'Get Data' (on the left side of the window) and select bed as file format and Human Feb. 2009 (GRCh37/hg19) as assembly version. The uploaded file will appear on the right side of the page.

- On the left side, select 'NGS: Peak Annotation' under 'NGS TOOLBOX'. A number of tools will appear. Choose 'Genomic annotation of Chip-Seq peaks'. A new menu will appear. Use default parameters and click on 'Execute'. The results files (text and image) will appear on the right side.

For the following part, one has to previously run ChIP-Peak with different tag thresholds and save the peak lists under the following names (the 3-letter extension of the file names reflects the format):
stat1_t12_hg19.fps, stat1_t25_hg19.fps, stat1_t50_hg19.fps, stat1_t100_hg19.fps

stat1_t25_hg19.sga

Figure 5. Motif enrichment analysis. (a) STAT1 consensus sequence (TTCNNNGAA) enrichment in peak lists obtained at various tag thresholds. **(b)** Comparisons of peak lists derived with ChIP-Peak versus peak lists published by ENCODE. **(c)** Comparative evaluation of three alternative STAT1 binding motif descriptions: (i) consensus sequence TTCNNNGAA, (ii) PWM from JASPAR and (iii) MEME-ChIP-derived PWM from the peak regions identified by ChIP-Peak (tag threshold 100).

To generate a motif enrichment plot for the peak region +/-500bp around the center, go to:

http://ccg.vital-it.ch/ssa/oprof.php

Fill out the input form as shown in Table 3, and click on the Submit button.

SSA Input Data	Signal Description
**Upload custom Data**<br>Format: FPS, File: stat1_t12_hg19.fps<br>Experiment: STAT1 ChIP-Peak<br>Genome: H.sapiens (Feb 2009/hg19)<br>**Sequence Range**<br>Entire sequence range: unchecked<br>5'border: -499 3'border: 500<br>**Sliding window parameters**<br>Window size: 100<br>Window shift: 5<br>Search mode: bidirectional	**Consensus seq**<br>TTCNNNGAA<br>Mismatches: 0<br><br>Name: TTCNNNGAA<br>Reference Position: 5

Table 3. Motif studies with OProf.

Repeat the same type of analysis with the peak lists obtained with tag thresholds 25, 50 and 100. The combined results are shown in Figure 6a. Results from OProf can be saved in textual format under the names:

stat1_tr-12.dat, stat1_tr-25.dat, stat1_tr-50.dat, stat1_tr-100.dat

To generate the motif enrichment profiles shown if Figure 6b, select the following STAT1 peak lists from the ENCODE consortium, one from HeLa and one from K562 cell lines (*Wang et al., 2012*), under the menu heading 'Select available Data Sets':

- Genome: H. sapiens (Feb 2009/hg19)
- Data type: ENCODE ChIP-seq-peak
- Series: Wang et al. 2012, Transcription Factor Binding Sites from ENCODE
- Sample: Hela-S3 STAT1 std - IFNg30 - peaks

Carry out the same type of analysis as described in Table 3, and save the results in text files named respectively:
Hela-Stat1_encode.dat, and K562-Stat1_encode.dat

To search the JASPAR MA0137.3 STAT1 motif (Figure 3c), fill out the OProf input form following the instructions given in Table 4, and click Submit.

SSA Input Data	Signal Description
**Upload custom Data**<br>Format: FPS, File: stat1_t50_hg19.fps<br>Experiment: STAT1 ChIP-Peak<br>Genome: H.sapiens (Feb 2009/hg19)<br>**Sequence Range**<br>Entire sequence range: unchecked<br>5'border: -499 3'border: 500<br>**Sliding window parameters**<br>Window size: 100 Window shift: 5<br>Search mode: bidirectional	**PWMs from Library**<br>Motif Library: JASPAR CORE 2014 vertebrates<br>Motif: STAT1 MA0137.3 (length=11)<br><br>Cut-off: p-value<br>Value: 0.0001<br><br>Name: MA0137.3 STAT1<br>Reference Position: 6

Table 4. PWM profile with OProf.

Save the results as:

stat1_jaspar2014_tr-50.dat

To search the MEME-derived motif (Figure 3c), we use the MEME-ChIP server (*Machanick and Bailey, 2011*). The sequence file provided as input to the MEME-ChIP server can be generated using the sequence extraction tool on the ChIP-Peak output page. Use the repeat-masked peak list obtained with tag threshold 100. Extract sequences from position −60 to +60 relative to the peak centers, using the bottom-left menu on the ChIP-Peak result page. This returns a list of sequences around the peaks in FASTA format. A new link 'Sequence File' will appear. Open this link. Now, open the home page of the **MEME** Suite in another browser window:

1. http://meme.sdsc.edu

and click on the **MEME-Chip** link near the lower right corner of the page. Enter your email address, then copy&paste the sequences from the open **ChIP-Peak** window into the text area of the **MEME-ChIP** page that serves for sequence input. Since we expect the STAT1 binding motif to be palindromic, we restrict the **MEME-ChIP** search to palindromic motifs.
Keep all the default parameters except the maximum motif width (=15), and check the box near 'look for palindromes only'.
On the **MEME-ChIP** result page, click on the link 'MEME-ChIP html output' to get the result page.
Here is the main motif found by MEME:

E-value 1.2e-803<br>Width 11<br>Sites 553	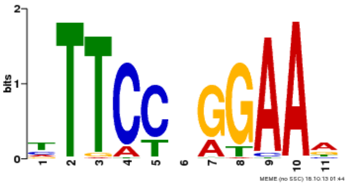

You can expand all clusters to show all motifs. Besides the graphical display of motif 1, you can click on the link “MEME” to view the MEME output. At the “Discovered Motifs” section, besides the logo of motif 1, you click on the arrow “Submit/Download” and a pop-up window will appear allowing you to download the motif. There, click on "Download Motif" and select the “Minimal MEME” format. Both a probability and a log-odds matrices will appear. The ‘log-odds’ matrix scoring is the following:

```
 -89      5    -96     92
-656   -438   -462    193
-468   -607   -303    190
-100    180   -549   -414
-369    163   -508    -22
  10    -11    -11     10
 -22   -508    163   -369
-414   -549    180   -100
 190   -303   -608   -468
 193   -462   -438   -656
  92    -96      5    -89
```

Keep this window open and fill out the OProf input form following the instructions given in Table 5.

SSA Input Data	Signal Description
**Upload custom Data**<br>Format: FPS, File: stat1_t50_hg19.fps<br>Experiment: STAT1 ChIP-Peak<br>Genome: H.sapiens (Feb 2009/hg19)	**Custom Weight Matrix**<br>Format: Integer PWM<br>PWM text: cut&paste matrix from MEME output into text area
**Sequence Range**<br>Entire sequence range: unchecked<br>5’border: -499 3’border: 500	Cut-off: p-value<br>Value: 0.00015
**Sliding window parameters**<br>Window size: 100 Window shift: 5<br>Search mode: bidirectional	Name: MEME-ChIP motif<br>Reference Position: 6

Table 5. PWM profile with OProf.

Save the results as:

stat1_MEME-ChIP_tr-50.dat

R Code to generate the Figure

```
----------------------------------------------------------------------------------------------------------------------------

stat1.t12 <- read.table("stat1_tr-12.dat")
stat1.t25 <- read.table("stat1_tr-25.dat")
stat1.t50 <- read.table("stat1_tr-50.dat") #pval : 0.0001
stat1.t100 <- read.table("stat1_tr-100.dat")
stat1.jaspar.t50 <- read.table("stat1_jaspar2014_tr-50.dat") #pval : 0.0001
stat1.MEME.t50 <- read.table("stat1_MEME-ChIP_t50.dat"); #pval : 0.00015
jpeg("Fig6_STAT1_motifs_plots.jpg", width = 3000, height = 1000, quality = 100, pointsize=35)
```

```
par(mfrow=c(1,3))
plot(stat1.t100, type="l", frame.plot=F, ylim=c(0,100), xlab="Distance to STAT1 peaks centers",
ylab="Frequency (%)", main="", lwd=7, xlim=c(-500,500), cex.axis=1.7, cex.lab=1.5)
points(stat1.t50, type="l", col="darkred", lty=2, lwd=7)
points(stat1.t25, type="l", col="blue", lty=3, lwd=7)
points(stat1.t12, type="l", col="skyblue", lty=4, lwd=7)
legend("topleft", legend=c("ChIP-Peak(th-100; 1521 peaks)","ChIP-Peak(th-50; 4443 peaks)","ChIP-
Peak(th-25; 16332 peaks)","ChIP-Peak(th-12; 55921 peaks)"), lty=c(1,2,3,4),
col=c("black","darkred","blue","skyblue"), bty="n", lwd=5, cex=1.8)
text(x=-530, y=112, labels=expression(paste(bold("(a)"))), xpd=NA, cex=3)
plot(stat1.t100, type="l", frame.plot=F, ylim=c(0,100), xlab="Distance to STAT1 peaks centers",
ylab="Frequency (%)", main="", lwd=7, xlim=c(-500,500), cex.axis=1.7, cex.lab=1.5)
points(stat1.t50, type="l", col="darkred", lty=2, lwd=7)
points(stat1.Hela, type="l", col="green", lty=5, lwd=7)
points(stat1.K562, type="l", col="darkgreen", lty=6, lwd=7)
legend("topleft", legend=c("ChIP-Peak(th-100; 1521 peaks)","ChIP-Peak(th-50; 4443
peaks)","ENCODE (K562; 890 peaks)","ENCODE (HeLa-S3; 5629 peaks)"), lty=c(1,2,6,5),
col=c("black","darkred","darkgreen","green"), bty="n", lwd=5, cex=1.8)
text(x=-530, y=112, labels=expression(paste(bold("(b)"))), xpd=NA, cex=3)
plot(stat1.jaspar.t50, type="l", frame.plot=F, ylim=c(0,100), xlab="Distance to STAT1 peaks centers",
ylab="Frequency (%)", main="", lwd=7, xlim=c(-500,500), cex.axis=1.7, cex.lab=1.5)
points(stat1.MEME.t50, type="l", col="blue", lty=2, lwd=7)
points(stat1.t50, type="l", col="darkred", lty=3, lwd=7)
legend("topleft", legend=c("JASPAR MA0137.3 motif", "MEME-ChIP motif", "STAT1 consensus seq"),
lty=c(1,2,3), col=c("black", "blue", "darkred"), bty="n", lwd=5, cex=1.8)
text(x=-530, y=112, labels=expression(paste(bold("(c)"))), xpd=NA, cex=3)
dev.off()
-----------------------------------------------------------------------------------------------------------------------------------
```

Figure 6. Histone modifications around STAT1 peaks. (a) Distribution of three histone marks around STAT1 peaks from interferon-γ stimulated HeLa cells. Note that the histone marks have been assayed in non-stimulated Hela cells where STAT1 is not supposed to bind to any of its genomic target sites. **(b)** H3K27ac marks around STAT1 peaks in HeLa and other cell types.

Open ChIP-Cor, and fill out the input form as detailed in Table 6. Rerun ChIP-Cor with the corresponding samples for H3K27ac and H3K27me3. Use 70 bp as the centering distance, as it would correspond to the average nucleosome DNA length (of about 140 bp).

ChIP-Seq Input Data Reference Feature	ChIP-Seq Input Data Target Feature
**Upload Custom Data**<br>Format: SGA<br>File: stat1_t25_hg19.sga<br>Genomes: H. sapiens (Feb 2009/hg19)	**Select available Data Sets**<br>Genome: H. sapiens (Feb 2009/hg19)<br>Data type: ENCODE ChIP-seq<br>Series: GSE29611, Histone Modifications by ChIP-seq<br>Sample: Hela-S3 H3K4me3
**Additional Input Data Options**<br>Strand: any<br>**Analysis Parameters**<br>Range: -5000 to 5000<br>**Histogram Parameters**<br>Window width: 10<br>Count Cut-off: 1<br>Normalization: global	**Additional Input Data Options**<br>Strand: any<br>Centering: 70

Table 6. Histone modification profiles with ChIP-Cor.

Save the results from the three runs under the following file names:

robertson_stat1_H3K4me3.out,robertson_stat1_H3K27ac.out, robertson_stat1_H3K27me3.out

For H3K27ac mark profiles in HeLa and other cell types (Figure 7b), repeat the step-by-step procedure in Table 6 with the following samples:

- 'H1-hESC H3K27ac', 'K562 H3K27me3'

Save the results as:

robertson_stat1_H3K27ac_K562.out, robertson_stat1_H3K27ac_H1-hESC.out

R Code to generate the Figure

```
------------------------------------------------------------------------------------------------------------------------------------
stat1p.h3k4me3 <- read.table("robertson_stat1_H3K4me3.out")
stat1p.h3k27ac <- read.table("robertson_stat1_H3K27ac.out")
stat1p.h3k27me3 <- read.table("robertson_stat1_H3K27me3.out")

stat1p.h3k27ac.k562 <- read.table("robertson_stat1_H3K27ac_K562.out")
stat1p.h3k27ac.h1 <- read.table("robertson_stat1_H3K27ac_H1-hESC.out")
jpeg("Fig7_STAT1_HistoneMod", width = 2000, height = 1000, quality = 100, pointsize=28)
par(mfrow=c(1,2))
plot(stat1p.h3k27ac, type="l", frame.plot=F, ylim=c(0,50), xlab="Distance to STAT1 peaks centers",
ylab="Fold Enrichment", lwd=7, cex.axis=1.5, cex.lab=1.3)
points(stat1p.h3k4me3, type="l", col="blue", lty=2, lwd=7)
points(stat1p.h3k27me3, type="l", col="darkred", lty=3, lwd=7)
text(x=-5400, y=57, labels=expression(paste(bold("(a)"))), xpd=NA, cex=2.3)
legend("topright", legend=c("H3K27ac (HeLa-S3)","H3K27me3 (HeLa-S3)","H3K4me3 (HeLa-S3)"),
lty=c(1,2,3), col=c("black","darkred","blue"), bty="n", lwd=5, cex=1.3)
plot(stat1p.h3k27ac, type="l", frame.plot=F, ylim=c(0,50), xlab="Distance to STAT1 peaks centers",
ylab="Fold Enrichment", main="", lwd=7, cex.axis=1.5, cex.lab=1.3)
points(stat1p.h3k27ac.k562, type="l", col="darkred", lty=2, lwd=7)
points(stat1p.h3k27ac.h1, type="l", col="blue", lty=3, lwd=7)
text(x=-5400, y=57, labels=expression(paste(bold("(b)"))), xpd=NA, cex=2.3)
legend("topright", legend=c("H3K27ac - HeLa-S3","H3K27ac - K562","H3K27ac - H1-hESC"),
lty=c(1,2,3), col=c("black","darkred","blue"), bty="n", lwd=5, cex=1.3)
dev.off()
------------------------------------------------------------------------------------------------------------------------------------
```

Figure S2. DNase I hypersensitivity, sequence conservation and population variation near STAT1 sites. (a) DNAse I hypersensitivity around STAT1 peaks. **(b)** PhastCons conservation scores and population variation around STAT1 peaks.

To explore DNase I hypersensitivity near STAT1 sites in the same three cell types (Figure S2a), choose the following samples as target features:

- Genome: H. sapiens /Feb 2009/hg19
- Data type: ENCODE DNAse FAIRE etc.
- Series: Thurman 2012, DNaseI Hyper-sensitivity by Digital DNaseI...
- Sample: DNaseI HS - Hela-S3 - None - Rep1

and carry out the analysis with the same parameters as shown in Table 6 except:

- Range: -1000 to 1000

• Centering: (leave blank for target feature)

Save the results under the following names:

robertson_stat1_DNaseI_HeLa.out,robertson_stat1_DNaseI_K562.out, robertson_stat1_DNaseI_H1-hESC.out

For cross-species conservation around STAT1 peaks (Figure S2a), use the phastCons conservation scores from UCSC, and fill out the ChIP-Cor input form as shown in Table 7.

ChIP-Seq Input Data Reference Feature	ChIP-Seq Input Data Target Feature
**Upload Custom Data**<br>Format: FPS<br>File: stat1_t25_rmsk_hg19_sites.fps<br>Genomes: H. sapiens (Feb 2009/hg19)<br>**Additional Input Data Options**<br>Strand: oriented<br>**Analysis Parameters**<br>Range: -1000 to 1000<br>**Histogram Parameters**<br>Window width: 10<br>Count Cut-off: 10<br>Normalization: global	**Select available Data Sets**<br>Genome: H. sapiens (Feb 2009/hg19)<br>Data type: Sequence-derived<br>Series: Vertebrate conservation (phastCons46way)<br>Sample: PHASTCONS VERT46<br>**Additional Input Data Options**<br>Strand: any<br>Repeat Masker: unchecked

Table 7. PhastCons conservation scores with ChIP-Cor.

For population studies (Figure S2b), select the following data sets as target features:

- Series: SNP collection from 1000 genome project
- Sample: Common SNPs
- Sample: All indels

and repeat the analysis with the same parameters as shown in Table 7.

Save the results under the following file names:
stat1_t25_hg19_PhastCons_foldenrich.out,stat1_t25_indels_1000GP_foldenrich.out, stat1_t25_hg19_commonSNPs_1000GP_foldenrich.out

R Code to generate the Figure

```
---------------------------------------------------------------------------------------------------------------------------

stat1p.dnase1 <- read.table("robertson_stat1_DNaseI_HeLa.out")
stat1p.dnase1.k562 <- read.table("robertson_stat1_DNaseI_K562.out")
stat1p.dnase1.h1 <- read.table("robertson_stat1_DNaseI_H1-hESC.out")
stat1p.dnase1.GM12878 <- read.table("robertson_stat1_DNaseI_GM12878.out")
stat1.t25.PhastCons <- read.table("stat1_t25_hg19_PhastCons_foldenrich.out")
stat1.t25.indels <- read.table("stat1_t25_indels_1000GP_foldenrich.out")
stat1.t25.SNPs <- read.table("stat1_t25_hg19_commonSNPs_1000GP_foldenrich.out")
jpeg("FigS2_DNase_ConsVar.jpg", width = 2000, height = 1000, quality = 100, pointsize=32)
par(mfrow=c(1,2))
plot(stat1p.dnase1, type="l", frame.plot=F, ylim=c(0,70), xlab="Distance to STAT1 peaks centers",
ylab="Fold Enrichment", main="", lwd=4, cex.axis=1.2, cex.lab=1.2)
points(stat1p.dnase1.k562, type="l", col="darkred", lty=2, lwd=4)
points(stat1p.dnase1.h1, type="l", col="blue", lty=3, lwd=4)
text(x=-1050, y=77, labels=expression(paste(bold("(a)"))), xpd=NA, cex=1.8)
legend("topleft", legend=c("DNase I HS - HeLa-S3","DNase I HS - K562","DNase I HS - H1-hESC"),
lty=c(1,2,3), col=c("black","darkred","blue"), bty="n", lwd=4)
```

```
plot(stat1.t25.PhastCons , frame.plot="F", xlab="Distance to STAT1 peak centers", ylab="Fold
Enrichment", col="black", lwd=7, type="l", ylim=c(0,3), xlim=c(-1000,1000), xaxt="n", yaxt="n",
cex.axis=1.2, cex.lab=1.2)
axis(1, at = seq(-1000,1000,500), lwd = 2, las=1, cex.axis=1.2)
axis(2, at = seq(0,3,1), lwd = 2, las=1, cex.axis=1.6)
text(x=-1050, y=3.30, labels=expression(paste(bold("(b)"))), xpd=NA, cex=1.8)
points(stat1.t25.indels, type="l", col="darkred", lty=2, lwd=4)
points(stat1.t25.SNPs, type="l", col="blue", lty=3, lwd=4)
legend("topleft", legend=c("PhastCons Vert46","Indels (1000 GP)","Common SNPs (1000 GP)"),
lty=c(1,2,3), col=c("black","darkred","blue"), bty="n", lwd=4)
dev.off()
```

Figure 7. High resolution aggregation plots for in-vivo occupied STAT1 sites (a) Single-base resolution PhyloP profile around STAT1 motifs aligned with the sequence Logo of the JASPAR STAT1 matrix. The motif sequence logo has been superimposed using GIMP. **(b)** Occurrence and distance preference of a second STAT1 motif downstream of an *in vivo* bound motif.

To look at cross-species conservation of the in vivo bound STAT1 sites using the PhyloP base-wise conservation scores from UCSC (Figure 8a), fill out the ChIP-Cor input form as shown in Table 8, submit it, and subsequently repeat the same procedure for the control set.

ChIP-Seq Input Data Reference Feature	ChIP-Seq Input Data Target Feature
**Upload Custom Data**<br>Format: FPS<br>File: stat1_t25_rmsk_hg19_sites.fps<br>Genomes: H. sapiens (Feb 2009/hg19)<br>**Additional Input Data Options**<br>Strand: oriented<br>**Analysis Parameters**<br>Range: -12 to 12<br>**Histogram Parameters**<br>Window width: 1<br>Count Cut-off: 10<br>Normalization: global	**Select available Data Sets**<br>Genome: H. sapiens (Feb 2009/hg19)<br>Data type: Sequence-derived<br>Series: PhyloP base-wise conservation<br>Sample: PhyloP vertebrate 46way (score ≥ 2)<br>**Additional Input Data Options**<br>Strand: any<br>Repeat Masker: unchecked

Table 8. PhyloP conservation scores with ChIP-Cor.

Save the results as:
stat1_phyloP_score_zoom.out, stat1_phyloP_control_zoom.out

The SSA program FindM can be used to compile a list of motifs located in the vicinity of peak center positions. To do so, go to the web form at:

http://ccg.vital-it.ch/ssa/findm.php

fill in the parameters as shown in Table 9, and click on the Submit button.

SSA Input Data	Signal Description
**Upload custom Data**<br>Format: FPS, File: stat1_t25_rmsk_hg19.fps<br>Experiment: Custom FPS<br>Genome: H.sapiens (Feb 2009/hg19)<br>**Sequence Range**<br>Entire sequence range: unchecked<br>5'border: -60 3'border: 60	**PWMs from Library**<br>Motif Library: JASPAR CORE 2014 vertebrates<br>Motif: STAT1 MA0137.3 (length=11)<br><br>Cut-off: p-value<br>Value: 0.0001<br><br>Name: MA0137.3 STAT1

**Sliding window parameters**<br>Search mode: forward<br>Sequence selection mode: best matches	Reference Position: 6

Table 9. Extract occupied sites with FindM.

Save the list of occupied binding motifs as:
stat1_t25_rmsk_hg19_sites.fps

For obtaining the control list (stat1_t25_rmsk_hg19_control.fps), repeat the above procedure changing the Sequence Range to 5'border: +10000, 3'border: +12000.

To search the MEME-derived motif around STAT1 sites (Figure 8b), fill out the OProf input form following the instructions given in Table 10, and repeat the same procedure for the control set.

SSA Input Data	Signal Description
**Upload custom Data**<br>Format: FPS, File: stat1_t25_rmsk_sites.fps<br>Experiment: STAT1 in-vivo<br>Genome: H.sapiens (Feb 2009/hg19)<br>**Sequence Range**<br>Entire sequence range: unchecked<br>5'border: 0 3'border: 100<br>**Sliding window parameters**<br>Window size: 13 Window shift: 1<br>Search mode: bidirectional	**Custom Weight Matrix**<br>Format: Integer PWM<br>PWM text: cut&paste matrix from MEME output into text area<br><br>Cut-off: p-value<br>Value: 0.001<br><br>Name: MEME-ChIP motif<br>Reference Position: 6

Table 10. MEME motif occurrence profile using OProf.

Save the results under the following file names:
MEME_motif1_co635_stat1_t25_hg19_rmsk_sites.out,
MEME_motif1_co635_stat1_t25_hg19_rmsk_control.out

R Code to generate the Figure

```
phylop.stat1.zoom <- read.table("stat1_phyloP_score_zoom.out")
phylop.ctrl.zoom <- read.table("stat1_phyloP_control_zoom.out")
phylop.stat1.zoom.mod <- as.data.frame(matrix(NA,ncol=2, nrow=(dim(phylop.stat1.zoom)[1]-1)))
phylop.ctrl.zoom.mod <- as.data.frame(matrix(NA,ncol=2, nrow=(dim(phylop.ctrl.zoom)[1]-1)))
for (i in 1:(dim(phylop.stat1.zoom)[1]-1)){
  phylop.stat1.zoom.mod[(i*2)-1,] <- phylop.stat1.zoom[i,]-c(0.5,0)
  phylop.stat1.zoom.mod[i*2,1] <- phylop.stat1.zoom[i+1,1]-0.5
  phylop.stat1.zoom.mod[i*2,2] <- phylop.stat1.zoom[i,2]
}
for (i in 1:(dim(phylop.ctrl.zoom)[1]-1)){
  phylop.ctrl.zoom.mod[(i*2)-1,] <- phylop.ctrl.zoom[i,]-c(0.5,0)
  phylop.ctrl.zoom.mod[i*2,1] <- phylop.ctrl.zoom[i+1,1]-0.5
  phylop.ctrl.zoom.mod[i*2,2] <- phylop.ctrl.zoom[i,2]
}
jpeg("Fig8_STAT1_phyloP_MEMEprof.jpg", width = 2000, height = 1000, quality = 100, pointsize=32)
par(mfrow=c(1,2))
```

```
plot(phylop.stat1.zoom.mod, type="l", frame.plot=F, ylim=c(0,10), xlab="Distance to STAT1/Control
sites", ylab="Fold Enrichment", main="", lwd=5, cex.axis=1.2, cex.lab=1.2, xaxt="n")
axis(1, at=seq(-12, 12, 4), cex.axis=1.2)
points(phylop.ctrl.zoom.mod, type="l", col="darkred", lty=3, lwd=5)
text(x=-13.3, y=11.2, labels=expression(paste(bold("(a)"))), xpd=NA, cex=1.8)
legend("topleft", legend=c("PhyloP (STAT1 sites)","PhyloP (Control sites)"), lty=c(1,3),
col=c("black","darkred","blue"), bty="n", lwd=4,, cex=1.0)
MEME.motifs.stat1.sites <- read.table("MEME_motif1_co635_stat1_t25_hg19_rmsk_sites.dat")
MEME.motifs.stat1.control <- read.table("MEME_motif1_co635_stat1_t25_hg19_rmsk_control.dat")
plot(MEME.motifs.stat1.sites, frame.plot="F", xlab="Distance to STAT1/Control sites",
ylab="Frequency (%)", col="black", lwd=5, type="l", ylim=c(0,6), xlim=c(0,100), xaxt="n", yaxt="n",
cex.axis=1.2, cex.main=1.2, cex.lab=1.2, cex.sub=1.2)
axis(1, at = seq(0,100,20), lwd = 2, las=1, cex.axis=1.2)
axis(2, at = seq(0,6, 1), lwd = 2,  las=1, cex.axis=1.2)
points(MEME.motifs.stat1.control, type="l", col="darkred", lty=1, lwd=5)
text(x=-3.8, y=6.7, labels=expression(paste(bold("(b)"))), xpd=NA, cex=1.8)
legend(x="topleft", legend=c("Occurrence of MEME motif in occupied STAT1 sites", "Occurrence of
MEME-ChIP motif in Control set"), col=c("black", "darkred"), lty=c(1,1), bty="n", lwd=4, cex=0.8)
dev.off()
```

Table S1. ChIP-Seq command line programs:

Program Name	Description	Input format	Output Format	Language
**Correlation Tools**				
**chipcor**	Correlates two features corresponding to two ChIP-seq tag (or read) distributions	SGA	Text (histogram)	C
**chipextract**	Extracts target features tags that fall into a distance range relative to a reference feature	SGA	Text (heatmap)	C
**chipscore**	Extracts ChIP-seq tags from feature A that are enriched (or depleted) in feature B tags	SGA	SGA	C
**Peak finding Tools**				
**chippeak**	Locates ChIP-seq signal peaks	SGA	SGA	C
**chippart**	Partitions the genome into signal-rich and signal-poor segments	SGA	Special SGA	C
**Pre-processing Tools**				
**chipcenter**	Shifts ChIP-seq tags to estimated center positions of DNA fragments	SGA	SGA	C
**counts_filter**	Filters out or select all ChIP-seq tags that occur within a set of DNA regions (e.g. repeat mask)	SGA	SGA	C
**Reformatting Tools**				
**compactsga**	Merges equal ChIP-seq tag positions into a single line adjusting the count field	SGA	SGA	C
**featreplace**	Changes the name of the <feature> field	SGA	SGA	C
**Perl conversion Tools**				
**bed2sga.pl**	Converts BED format into SGA format	BED	SGA	Perl
**fps2sga.pl**	Converts FPS format into SGA format	FPS	SGA	Perl
**gff2sga.pl**	Converts GFF format into SGA format	GFF	SGA	Perl
**partit2sga.pl**	Converts the output from the chippart tool into centered SGA format	Special SGA	BED	Perl
**partit2bed.pl**	Converts the output from the chippart tool into BED format	Special SGA	BED	Perl
**partit2gff.pl**	Converts the output from the chippart tool into GFF format	Special SGA	GFF	Perl
**wigVS2sga.pl**	Converts Wig Variable Step format into SGA format	Wig	SGA	
**sga2bed.pl**	Converts SGA format into BED format	SGA	BED	Perl
**sga2gff.pl**	Converts SGA format into GFF format	SGA	GFF	Perl
**sga2fps.pl**	Converts SGA format into FPS format	SGA	FPS	Perl
**sga2wigVS.pl**	Converts SGA format into Wig Variable Step format	SGA	Wig	Perl
**sga2wigFS.pl**	Converts SGA format into Wig Fixed Step format	SGA	Wig	Perl

Table S2. Contents of the MGA repository

Data Type	Human	Mouse	Fruit Fly	Worm	Zebra fish	Baker's Yeast	Arabidopsis	Fission Yeast	Corn	Bee	Total
**ChIP-seq**	6227	616	230	6	16	25	32	57	8	-	7217
**ChIP-seq-peak**	1683	26	-	-	-	-	-	-	-	-	1709
**RNA-seq**	1108	385	13	19	12	22	4	1	8	16	1588
**Dnase FAIRE etc.**	1034	10	23	6	4	27	-	8	-	-	1112
**DNA methylation**	24	4	-	-	-	-	-	-	-	-	28
**Genome annotation**	23	11	8	8	6	4	2	4	3	2	70
**Sequence-derived**	13	3	6	-	4	-	-	-	-	-	26
**Total # of Samples**	10113	1055	280	39	42	78	38	69	19	18	11751

Table S3. Survey of related resources

Property	Nebula[1]	Cistrome[2]	Galaxy Main[3]	GalaxEast[4]	ColoWeb[5]	GeneProf[6]	W-ChIPeaks[7]	HTSstation[8]	ChIP-Seq Tools[9]
**Data upload capabilities**									
File or URL	✔	✔	✔	✔	✔ (files)	✔ (+ SRA ID)	✔ (files)	✔	✔
Sequence (SRA, fastq, fasta)	✔	✔	✔	✔	.	✔	.	✔	.
Mapped reads (BAM, BED)	✔	✔	✔	✔	.	✔	✔	✔	✔
Genomic feature (BED, GFF, etc)	✔	✔	✔	✔	✔ (BED)	.	✔	.	✔
**Server resident data**									
Availability	.	✔	.	.	✔	✔	.	.	✔
Numbers	.	11949	.	.	10	2617	.	.	11515
Type	.	ChIP-Seq (Human & Mouse)	.	.	ChIP-Seq (Human)	ChIP-Seq, RNAseq (Human & Mouse)	.	.	Multiple data types, organisms, assemblies
**Analysis performed**									
Read mapping	✔ (Bowtie)	.	✔ (Bowtie, BWA)	✔ (Bowtie, BWA)	.	✔ (Bowtie)	.	✔ (Bowtie)	.
Peak finding and segmentation	✔ (MACS, FindPeaks, etc)	✔ (MACS, MACS2)	✔ (MACS, SICER)	✔ (MACS, MACS2, HOMER)	.	✔ (MACS)	✔ (in-house method)	✔ (MACS +in-house method)	✔ (in-house method)
Feature correlation via AP	.	✔	. (+/- via MACS)	. (+/- via MACS)	✔	.	.	.	✔
Heatmap	.	✔	. (custom plots)	✔ (via bigWig)	✔(limited: 200,000 features)	.	.	✔	✔
Motif analysis	✔ (ChIPmunk)	✔ (SeqPos)	.	.	.	.	.	✔ (Meme + in-house)	✔
Peak list annotation	✔	✔	.	✔	.	✔	.	✔	.
Feature selection*[1]	.	.	.	.	.	.	.	.	✔
Liftover files	.	✔	✔	.	.	.	.	.	✔
**Interoperability**									
Sequence extraction	✔	✔	✔	✔	.	.	.	✔	✔
Export BED output to other servers	✔	✔ (IGB & UCSC)	✔ (IGB)	✔ (UCSC)	.	. (not directly)	.	✔ (UCSC)	✔
Extract numerical results (AP, heatmap)	✔ (only statistics)	. (only AP)	.	. (only heatmap)	✔ (only statistics)	.	.	.	✔
UCSC tracks link / files	✔	✔	✔	✔	.	. (internal browser)	✔	.	✔
**Peak finding performance*[2]**									
Speed (min)	~10	~10	~5	>15	.	~15	~15	.	~1

*[1] Selection of items from genomic feature lists (e.g. TSSs, ChIP-seq peaks) based on colocalization criteria with another genomic feature (e.g. ChIP-seq tags, conservation scores)

*[2] Peak finding performance has been assessed using the STAT1 data set from (Robertson, et al., 2007), and the default web-based analysis parameters. The average number of obtained peaks is 40'000

1. https://nebula.curie.fr/
2. http://cistrome.org/ap/root
3. https://usegalaxy.org/
4. http://www.galaxeast.fr/
5. http://projects.insilico.us.com/ColoWeb/
6. http://www.geneprof.org/GeneProf/
7. http://compbio.uthscsa.edu/W-ChIPeaks/
8. http://htsstation.epfl.ch/
9. http://ccg.vital-it.ch/chipseq/

Figure S1. Peak fitting parameters (from ChIP-Cor)

```
          Single Gaussian Fit Parameters

          Formula: y ~ a + (b/(sig*sqrt(2*pi))) * exp(-(x - mean)^2/(2 * sig^2))

          Estim.  SE        t        Pr(>|t|)
a         0.00934 7.36e-05           127       2.43e-189
b         7.86    0.0906    86.7     1.07e-157
sig       143     1.65      87       5.63e-158
mean      0.507   1.5       0.338    0.736

          Residual =  0.01487

          Peak Finding Parameters Estimate

Av. BG density  (cnts)  0.00934
Peak window width (bp)  286
Peak threshold  (cnts)  12

          Peak Threshold    Expected Random Peaks
          8                 19437
          9                 5045
          10                1196
          11                261
          12                53
```

Figure S2. DNase I hypersensitivity, sequence conservation and population variation near STAT1 sites. (a) DNAse I hypersensitivity around STAT1 peaks. **(b)** PhastCons conservation scores and population variation around STAT1 peaks.

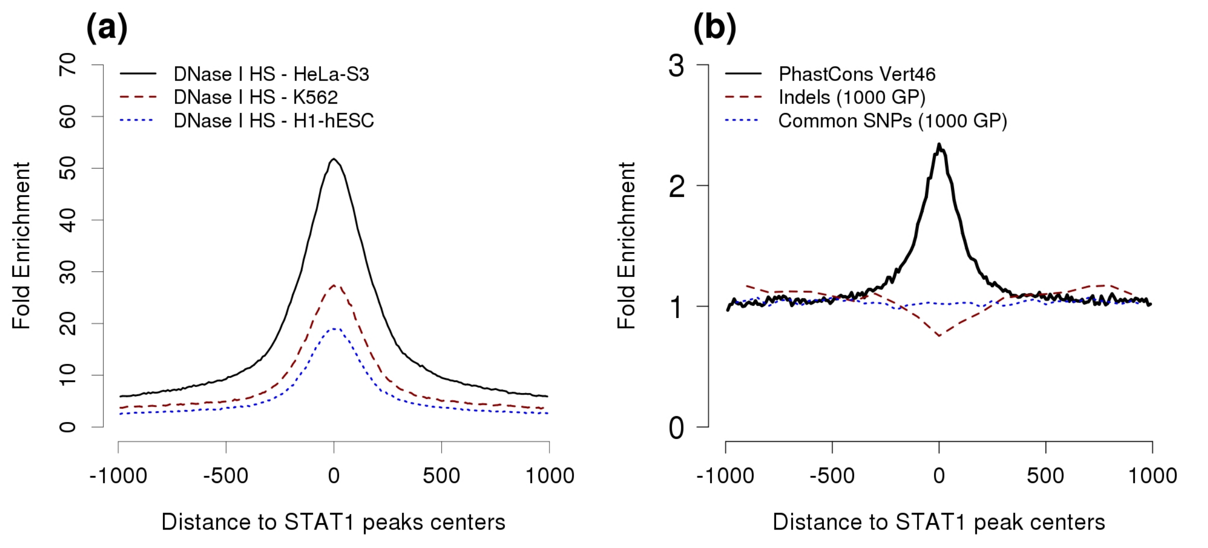

Supplement: Supplementary file 1 — Supplementary Material: Texts S1–S4, Figures S1–S2, Tables S1–S3. (PDF 1043 kb) [file 12864_2016_3288_MOESM1_ESM.pdf]
